# Genomic Multiple Sclerosis Risk Variants Modulate the Expression of the *ANKRD55*–*IL6ST* Gene Region in Immature Dendritic Cells

**DOI:** 10.3389/fimmu.2021.816930

**Published:** 2022-01-17

**Authors:** Jorge Mena, Iraide Alloza, Raquel Tulloch Navarro, Ane Aldekoa, Javier Díez García, Ane Villanueva Etxebarria, Cecilia Lindskog, Alfredo Antigüedad, Sabas Boyero, María del Mar Mendibe-Bilbao, Amaya Álvarez de Arcaya, José Luis Sánchez Menoyo, Luciana Midaglia, Noelia Villarrubia, Sunny Malhotra, Xavier Montalban, Luisa María Villar, Manuel Comabella, Koen Vandenbroeck

**Affiliations:** ^1^ Inflammation & Biomarkers Group, Biocruces-Bizkaia Health Research Institute, Barakaldo, Spain; ^2^ Microscopy Facility, Biocruces-Bizkaia Health Research Institute, Barakaldo, Spain; ^3^ Kronikgune Institute for Health Services Research, Barakaldo, Spain; ^4^ Health Service Research Network on Chronic Diseases Red de Investigación en Servicios de Salud en Enfermedades Crónicas (REDISSEC), Bizkaia, Spain; ^5^ Osakidetza-Basque Health Service, Research Unit, Galdakao University Hospital, Galdakao, Spain; ^6^ Department of Immunology, Genetics and Pathology, Rudbeck Laboratory, Uppsala University, Uppsala, Sweden; ^7^ Department of Neurology, Cruces University Hospital, Osakidetza-Basque Health Service, Biocruces-Bizkaia Health Research Institute, Barakaldo, Spain; ^8^ Department of Neurology, Txagorritxu University Hospital, Osakidetza-Basque Health Service, Bioaraba Health Research Institute, Vitoria-Gasteiz, Spain; ^9^ Department of Neurology, Galdakao-Usansolo University Hospital, Osakidetza-Basque Health Service, Biocruces-Bizkaia Health Research Institute, Galdakao, Spain; ^10^ Servei de Neurologia-Neuroimmunologia, Centre d’Esclerosi Múltiple de Catalunya (Cemcat), Institut de Recerca Vall d’Hebron (VHIR), Hospital Universitari Vall d’Hebron, Universitat Autònoma de Barcelona, Barcelona, Spain; ^11^ Department of Immunology, Hospital Universitario Ramón y Cajal, Instituto Ramón y Cajal de Investigación Sanitaria (IRYCIS), Red Española de Esclerosis Múltiple (REEM), Madrid, Spain; ^12^ Department of Biochemistry and Molecular Biology, Universidad del País Vasco (UPV/EHU), Barrio Sarriena, Leioa, Spain; ^13^ Ikerbasque, Basque Foundation for Science, Bilbao, Spain

**Keywords:** *ANKRD55*, *IL6ST*, sgp130, multiple sclerosis, autoimmune

## Abstract

Intronic single-nucleotide polymorphisms (SNPs) in the *ANKRD55* gene are associated with the risk for multiple sclerosis (MS) and rheumatoid arthritis by genome-wide association studies (GWAS). The risk alleles have been linked to higher expression levels of *ANKRD55* and the neighboring *IL6ST* (gp130) gene in CD4^+^ T lymphocytes of healthy controls. The biological function of ANKRD55, its role in the immune system, and cellular sources of expression other than lymphocytes remain uncharacterized. Here, we show that monocytes gain capacity to express *ANKRD55* during differentiation in immature monocyte-derived dendritic cells (moDCs) in the presence of interleukin (IL)-4/granulocyte-macrophage colony-stimulating factor (GM-CSF). *ANKRD55* expression levels are further enhanced by retinoic acid agonist AM580 but downregulated following maturation with interferon (IFN)-γ and lipopolysaccharide (LPS). *ANKRD55* was detected in the nucleus of moDC in nuclear speckles. We also analyzed the adjacent *IL6ST*, *IL31RA*, and *SLC38A9* genes. Of note, in healthy controls, MS risk SNP genotype influenced *ANKRD55* and *IL6ST* expression in immature moDC in opposite directions to that in CD4^+^ T cells. This effect was stronger for a partially correlated SNP, rs13186299, that is located, similar to the main MS risk SNPs, in an *ANKRD55* intron. Upon analysis in MS patients, the main GWAS MS risk SNP rs7731626 was associated with *ANKRD55* expression levels in CD4^+^ T cells. MoDC-specific *ANKRD55* and *IL6ST* mRNA levels showed significant differences according to the clinical form of the disease, but, in contrast to healthy controls, were not influenced by genotype. We also measured serum sgp130 levels, which were found to be higher in homozygotes of the protective allele of rs7731626. Our study characterizes *ANKRD55* expression in moDC and indicates monocyte-to-dendritic cell (Mo–DC) differentiation as a process potentially influenced by MS risk SNPs.

## Introduction

**Figure 1:**
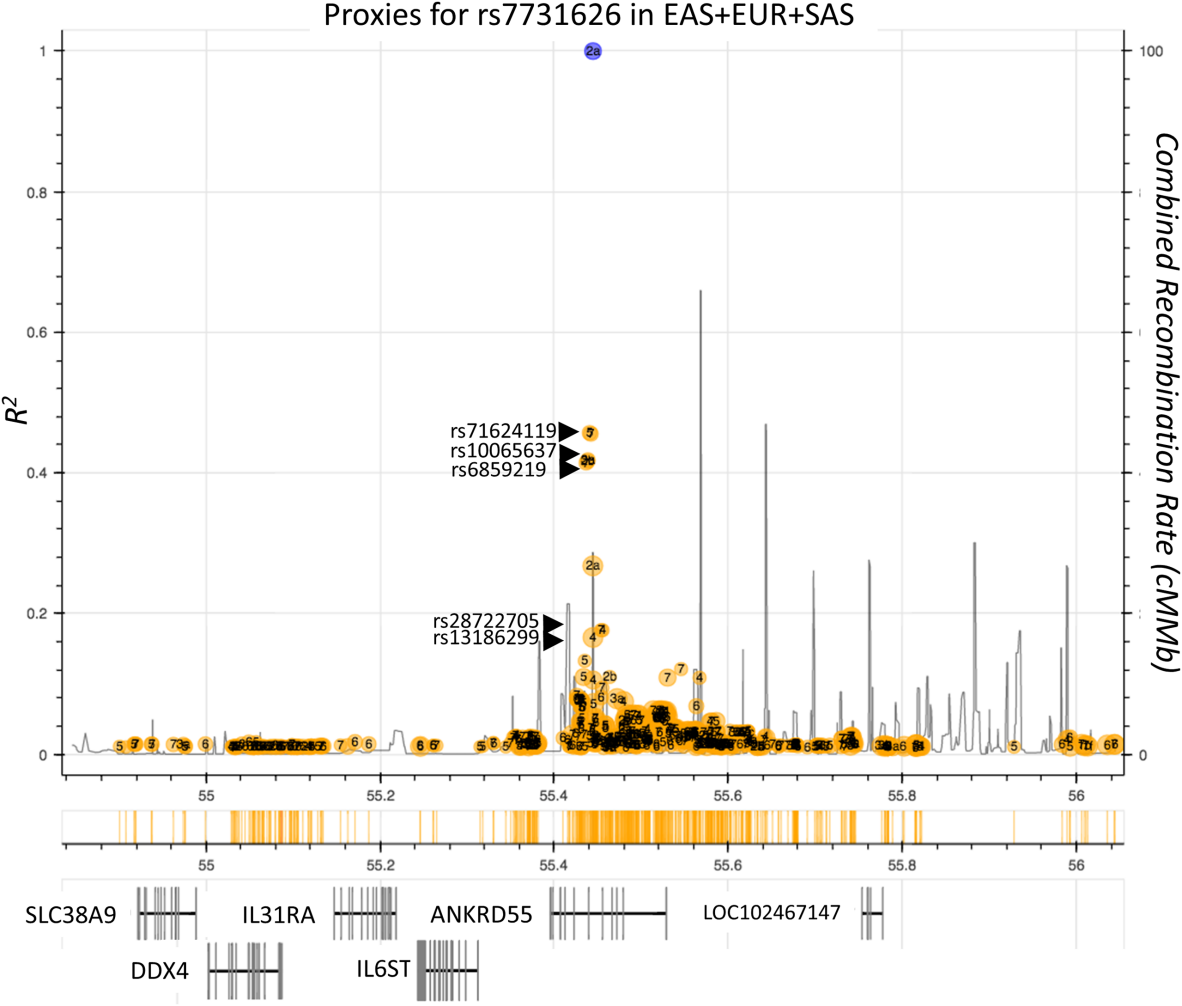
Autoimmune risk SNPs located in the *ANKRD55* gene area. Plot represents individual LD *r^2^* values of SNPs (ocher circles) located in the delineated genomic interval with the main autoimmune risk SNP rs7731626 (blue circle). SNPs independently associated with autoimmune diseases with genome-wide significance are indicated (rs71624119, rs10065637, rs6859219). Genes located in this interval are represented in the lower part of the figure. Combined recombination rate is provided (black lines). SNPs rs28722705 and rs13186299 have been associated robustly in GWAS with monocyte count (5, 6) and monocyte percentage of white cells (7), respectively. Image information was produced by means of ldlink-nci-nih-gov.

The *ANKRD55* gene, located on chromosome 5q11.2, contains single-nucleotide polymorphisms (SNPs) that emerged from genome-wide association studies (GWAS) as risk variants for disorders with predominantly autoimmune or chronic inflammatory etiopathogenesis. Among these SNPs, rs7731626 (Chr 5: 56148856) is the variant displaying the strongest association with both rheumatoid arthritis [RA; *P* < 2 × 10^-22^; ref (1, 2)] and multiple sclerosis [MS; *P* = 4 × 10^-15^; ref (3)]. This variant is also associated with pediatric autoimmune diseases (*P* = 1 × 10^-10^; ref (4). Other SNPs in *ANKRD55*, located like rs7731626 in intronic areas next to exon 6 (**Figure 1**), have, as well, been associated with the risk for autoimmune disease with genome-wide significance levels. Members of a trio of SNPs highly correlated among them, i.e., rs6859219 (Chr 5: 56142753), rs10065637, and rs71624119 (*D’* > 0.98; *R^2^* > 0.84 in European population), but more modestly to rs7731626 (*R^2^* < 0.53), were found to be associated with MS (8–10), RA (11–14), Crohn’s disease, and inflammatory bowel disease (15, 16). The intronic location of all these SNPs points to allelic variation in regulatory elements as potential mechanism for transcriptional modulation of *ANKRD55* and/or adjacent genes. Of these, several are immunologically relevant (*IL6ST* and the related receptor subunit *IL31RA*).

As *ANKRD55*’s proximate neighbor, *IL6ST* is especially striking due to its well-documented role in inflammation and autoimmune disease (**Figure 1**) (17). The *IL6ST* gene codes for the interleukin (IL)-6 receptor β unit, also known as glycoprotein 130 (gp130), which is part of the functional receptor of the pleiotropic cytokine IL-6 and is ubiquitously expressed. The gp130 chain forms also part of the receptor of other cytokines with demonstrated roles in autoimmune disease including IL-27 and IL-35 (17). Biological deregulation of IL-6 is functionally associated with chronic inflammation and autoimmunity (17). IL-6 can signal through two different pathways, i.e., classical signaling or trans-signaling, which mediate anti-inflammatory or pro-inflammatory response, respectively (17, 18). Distinct soluble forms of gp130 (sgp130) generated through alternative splicing or alternative polyadenylation, act as natural specific inhibitors of IL-6 trans-signaling; these include sgp130 (19), sgp130-RAPS (20), and sgp130-E10 (21). In MS, IL-6 blockage is thought to limit immune-mediated tissue damage. The IL-6 receptor blocking humanized monoclonal antibody (mAb) tocilizumab inhibits experimental autoimmune encephalomyelitis (EAE) (22) and is effective in otherwise refractory neuromyelitis optica spectrum disorder (NMOSD; ref (23, 24). Satralizumab, a second-generation anti-IL6R mAb, has been approved for the treatment of NMOSD (25).

The precise biological function of ANKRD55, on the other hand, is not yet known. Recent interactome and knockdown studies have provided some initial though heterogeneous indications. On the basis of integrated mass spectrometry protein interaction datasets, ANKRD55 was first identified as a novel member of the intraflagellar transport machinery (26). Correspondingly, morpholino knockdown of the gene appeared to disrupt ciliogenesis (26). In HEK293 cells, recombinant ANKRD55 interacts in the cytosol preferentially with 14-3-3 isoforms and ATP- or nucleotide-binding proteins (27). It appeared also capable of entering the nucleus of HEK293 cells where it is found preferentially associated with RNA-binding proteins and proteins involved in sumoylation (27). In preadipocytes, silencing of ANKRD55 by the lentiviral clustered regularly interspaced short palindromic repeats (CRISPR)/CRISPR-associated protein 9 (Cas9) system increased both their proliferation rate and lipolysis (28). A direct functional relevance of ANKRD55 to autoimmune processes has not been demonstrated.

*ANKRD55* was first reported as highly expressed in CD4^+^ effector memory cells (29). Peripheral blood CD4^+^ T lymphocytes, but not CD8^+^, CD19^+^, CD56^+^, or CD14^+^ subsets, express high levels of *ANKRD55* mRNA, and the risk allele of rs6859219 is associated with higher levels of *ANKRD55* mRNA in the CD4^+^ subset (30). This variant is associated with DNA methylation status at CpG sites mapping to *ANKRD55-IL6ST* intergenic and *ANKRD55* intronic areas. Increased methylation at these sites seen with the protective allele correlates with decreased expression of both *ANKRD55* and *IL6ST* mRNA in CD4^+^ T cells (31). The highly correlated rs71624119 variant was confirmed to colocalize to a *cis*-expression quantitative trait locus (eQTL) in CD4^+^ T cells that significantly affected the expression of *ANKRD55*. Evidence for shared effect of this SNP with an eQTL for *IL6ST* was weaker but still passed the false discovery rate (FDR) threshold (32). In a study in peripheral blood samples, both rs10065637 and rs6589219 colocalized with eQTLs for the expression of *ANKRD55* but not of *IL6ST* (33). Similarly, the Genotype-Tissue Expression (GTEx) Project lists the MS and RA lead risk variant rs7731626 as the SNP with the most significant *cis*-eQTL effect for the expression of *ANKRD55* in spleen and whole blood (GTEx Analysis Release V8)^^1^^. However, rs7731626 colocalized with CD4^+^ T-cell specific eQTLs for expression of both *ANKRD55* and *IL6ST*. This suggests that this locus has a pleiotropic mechanistic effect on transcription of the two genes in this specific cell subset (34). Risk for autoimmune disease conferred by rs7731626 is correlated with increased mRNA expression of *ANKRD55* and *IL6ST* and with reduced *cis* CpG methylation in CD4^+^ T cells (34, 35). Using naive CD4^+^ T-cell capture Hi-C data, a significant chromatin interaction between the intronic area around rs7731626 and the promoter of *IL6ST* was observed, potentially clarifying this coregulation in 3D space (31, 34, 36). On the whole, current evidence explains genetic risk for MS and RA conferred by *ANKRD55* SNPs in terms of a *cis* CpG methylation-dependent mechanism to increase the expression of two genes, *ANKRD55* and *IL6ST*, in CD4^+^ T lymphocytic cells.

Here, we show that CD14^+^ monocytes, which express only negligible levels of *ANKRD55* mRNA (30), gain capacity to produce higher levels of *ANKRD55* during differentiation into immature monocyte-derived dendritic cells (moDCs) in the presence of IL-4 and granulocyte-macrophage colony-stimulating factor (GM-CSF). *ANKRD55* levels are further enhanced by a retinoic acid agonist but are suppressed by interferon (IFN)-γ/lipopolysaccharide (LPS) treatment. We analyzed also neighboring genes including *IL6ST*, *IL31RA*, and *SLC38A9*. We demonstrate that in immature moDC, *ANKRD55* and *IL6ST* expression appears to be modulated by MS risk SNPs with an opposite allelic effect compared to that observed in CD4^+^ T lymphocytes.

## Materials and Methods

### Patients and Controls

Buffy coats and serum from healthy donors and MS patients’ whole blood were collected upon written informed consent in accordance with the local ethics committees from Bilbao [Comité Ético de Investigación Clínica de Euskadi (CEIC-E)], Barcelona (Comité Ético de Investigación Clínica Hospital Universitari Vall D’Hebron), and Madrid (Comité de Ética de la Investigación del Hospital Ramón y Cajal) correspondingly. All patients were under no treatment or placebo at the time of sample collection. A total of 128 biological samples of MS patients from 3 different Spanish cohorts (IIS Biocruces Bizkaia MS Cohort, Barakaldo; Hospital Universitari Vall d’Hebron MS Cohort, Barcelona; and Hospital Universitario Ramón y Cajal MS Cohort, Madrid) were included in the study [54 primary progressive (PP), 70 relapsing-remitting (RR), and 4 secondary progressive (SP)] in addition to 103 buffy coats from healthy control samples provided by the Biobanco Vasco. Patients’ demographic and clinical data are summarized in **Supplementary Table S1**.

### Peripheral Blood Mononuclear Cell Subpopulations

Peripheral blood mononuclear cells (PBMCs) were isolated from buffy coats using a Ficoll^®^-Paque density gradient (Cytiva, Ref. 17-1440-03) as indicated by the manufacturer. Fresh PBMCs were used for all experiments on monocytes and moDC. Comparative analysis of gene expression by qPCR was performed in CD4^+^, CD8^+^, CD14^+^, CD19^+^, and CD56^+^ cells purified from PBMC that had been stored in liquid nitrogen. CD4^+^ T-helper lymphocytes, CD8^+^ T cytotoxic lymphocytes, CD19^+^ B lymphocytes, CD14^+^ monocytes, and CD56^+^ natural killer (NK) cells were separated by positive selection using CD4 (Ref. 130-045-101), CD8 (Ref. 130-045-201), CD19 (Ref. 130-050-301), CD14 (Ref. 130-050-201), and CD56 (Ref. 130-050-401) human MicroBeads (all from Miltenyi Biotec) correspondingly, following the manufacturer’s instructions, as described before (30).

### Isolation of Myeloid Dendritic Cells and Plasmacytoid Dendritic Cells

Human myeloid DCs (mDCs) and plasmacytoid DCs (pDCs) were isolated from healthy donors’ fresh blood. PBMCs were obtained from 30 ml of blood by Ficoll^®^-Paque density gradient using SepMate™-50 tubes (STEMCELL Technologies, Ref. 85450). CD14^+^ monocytes were depleted from PBMCs using CD14 human MicroBeads (Miltenyi Biotec, Ref. 130-050-201). Remaining PBMCs were separated into two equal parts and processed with EasySep™ Human Myeloid DC Enrichment Kit (STEMCELL Technologies, Ref. 19061) for mDC isolation or EasySep™ Human Plasmacytoid DC Enrichment Kit (STEMCELL Technologies, Ref. 19062) for pDC isolation following the manufacturer’s protocol. To verify the purity of each subpopulation, cells were analyzed by flow cytometry (MACSQuant^®^ flow cytometer, Miltenyi Biotec) using anti-human CD1c (BCDA-1)-APC antibody (Miltenyi Biotec, Ref. 130-110-595) for mDC and CD303 (BDCA-2)-APC antibody (Miltenyi Biotec, Ref. 130-114-177) for pDC, as shown in **Supplementary Figure S1**. Isotype control human IgG1-PE antibody (Miltenyi Biotec, Ref. 130-113-428) was used as negative control for CD14 and isotype control human IgG1-APC as negative control for CD1c and CD303 (Miltenyi Biotec, Ref. 130-113-434).

### Sorting of Classical, Intermediate, and Non-Classical Monocytes

Monocytes were obtained from human PBMCs with Pan Monocyte Isolation kit (Miltenyi Biotec, Ref. 130-096-537) following the manufacturer’s protocol, labeled with anti-human CD14-PE antibody (Miltenyi Biotec, Ref. 130-110-519) and CD16-FITC (Miltenyi Biotec, Ref. 130-113-392), and sorted in a FACSJazz™ cell sorter (BD Biosciences). Sorter strategy consisted of a size-complexity selection of monocytes followed by discrimination of three monocyte subpopulations: classical (CD14^hi^/CD16^-^), intermediate (CD14^hi^/CD16^+^), and non-classical (CD14^+^/CD16^+^), as described in a previous article (37).

### Monocyte-Derived Dendritic Cell Differentiation and Maturation

CD14^+^ monocytes were isolated from fresh PBMCs by positive selection using CD14 MACS MicroBeads (Miltenyi Biotec, Ref. 130-050-201) and cultured at a density of 10^6^ cells/ml in moDC differentiation medium (containing IL-4/GM-CSF; Miltenyi Biotec, Ref. 130-094-812) for 6 days, with fresh medium added on day 3. Maturation of moDCs was induced using combinations of different agents, including LPS (0.2–2 µg/ml, as indicated) from *Salmonella* (Sigma-Aldrich, Ref. L6143), 0.5 pg/ml IFN-γ (Peprotech, Ref. AF-300-02), 1 μg/ml polyinosinic-polycytidylic acid (PolyI:C; Sigma-Aldrich, Ref. P1530), and 1 μg/ml oligodeoxyribonucleotide CpG (Miltenyi Biotec, Ref. 130-100-243). To induce tolerogenicity, DCs were treated during the differentiation process with 100 nM retinoic acid receptor-α agonist AM580 (Sigma-Aldrich, Ref. A8843), 10 nM vitamin D3 (STEMCELL Technologies, Ref. 72412), 1 μM prostaglandin E2 (PGE2) (Sigma-Aldrich, Ref. P0409), 20 ng/ml IL-10 (Peprotech, Ref. AF-200-10), 1 μM dexamethasone (Sigma-Aldrich, Ref. D4902), and 10 ng/ml rapamycin (STEMCELL Technologies, Ref. 73362), and all were added on day 0, and again on day 3, of the moDC differentiation period. For evaluation of maturation, cells were analyzed with Mo–DC Differentiation Inspector antibody cocktail [Miltenyi Biotec, Ref. 130-093-567; anti-human CD14-FITC antibody (clone Tük4, isotype: mouse IgG2a), anti-human CD83-APC (clone: HB15, isotype: mouse IgG1), and anti-human CD209-PE (clone DCN-47.5.4, isotype: mouse IgG1)] or isotype control cocktail following manufacturer’s instructions and analyzed on a MACSQuant^®^ flow cytometer (Miltenyi Biotec).

### DNA Extraction, RNA Isolation, and cDNA Synthesis

Genomic DNA (gDNA) was obtained from PBMCs with PureLink™ Genomic DNA purification kit (Invitrogen, Ref. K182001) and used for genotyping analyses. Total RNA was extracted from cells using TRI Reagent^®^ (Sigma-Aldrich, Ref. T9424), NucleoSpin RNA Kit (Macherey-Nagel, Ref. 740955), or RNeasy Mini Kit (Qiagen, Ref. 74104) following each manufacturer’s instructions, treated with DNAse (Sigma-Aldrich Ref. AMPD1 or Qiagen Ref. 79254) and measured by Nanodrop^®^ 2000 spectrophotometer (Thermo Fisher Scientific) to determine the concentration. cDNA was synthesized from 100 to 600 ng of total RNA using High-Capacity cDNA Reverse Transcription Kit (Applied Biosystems, Ref. 4368814).

### Primer Design, qPCR, Drop Digital PCR, and Genotyping

qPCR was performed using 5–30 ng cDNA, specific primer pairs (**Supplementary Table S2** **)** and Fast SYBR^®^ Green Master Mix (Applied Biosystems, Ref. 4385612) or SsoAdvanced™ Universal SYBR^®^ Green Supermix (Bio-Rad, Ref. 1725272), following manufacturer’s instructions, in a 7300, 7500 Fast or 7900HT Fast Real-Time PCR System (Applied Biosystems). *IL6ST* primers are best coverage primers that co-amplify the main splice variants of *IL6ST* by annealing to shared transcribed sequences (isoforms 206, 201, 205, 209, 204, 212, 203). *ACTB* and/or *GAPDH* was used as reference genes for normalization of mRNA expression level, and data were analyzed applying the 2^-ΔCt^ method. Samples were examined in triplicate for each condition, and no-template controls were included. The threshold was set within the linear phase of the logarithmic amplification plot. In specific experiments as stated in the text, absolute gene transcript quantification was performed by droplet digital PCR (ddPCR) from 350 pg or 30 ng of cDNA using ddPCR Supermix for Probes (No dUTP) (Bio-Rad, Ref. 1863023) and specific primer pairs (**Supplementary Table S2**) in a QX200 Droplet Digital PCR System (Bio-Rad) according to manufacturer’s protocol. gDNA Samples were genotyped for SNPs rs6859219, rs7731626, and rs13186299 using commercial TaqMan^®^ probes (Thermo Fisher Scientific, Ref. 4351379) and TaqPath™ ProAmp™ master mix (Applied Biosystems, Ref. A30865) in a 7500 Fast Real-Time PCR System (Applied Biosystems) following manufacturer’s instructions.

### Immunofluorescence

CD14^+^ monocytes were plated on coverslips coated with poly-D-lysine (Sigma-Aldrich, Ref. P0899) and differentiated for 6 days into moDC as mentioned above. Cells were fixed and permeabilized with ice-cold methanol for 5 min, blocked with 1% bovine serum albumin (BSA; Sigma-Aldrich, Ref. A9418) in phosphate buffered saline (PBS) for 30 min, stained with defined primary antibodies for 1 h at room temperature (RT), incubated with corresponding fluorescent secondary antibodies for 1 h at RT protected from light, stained with 1 μg/ml 4′,6-diamidino-2-phenylindole (DAPI) (Sigma-Aldrich, Ref. D9542) for 5 min at RT, and mounted on glass slides using Fluoromount-G (Invitrogen, Ref. 00-4958-02). Between each step, coverslips were washed 3 times with PBS. Primary antibodies used were anti-ANKRD55 rabbit polyclonal (Human Protein Atlas, Ref. HPA 061649; 1:250), anti-ALYREF mouse monoclonal (Thermo Fisher Scientific, Ref. MA1-26754; 1:500), anti-HNRNPC mouse monoclonal (Sigma-Aldrich, Ref. AMAB91010; 1:100), and anti-human CD209. Secondary antibodies used were AlexaFluor 488-conjugated goat anti-mouse IgG H&L (Abcam, Cat. No. ab150113; 1:1,000) and AlexaFluor 594-conjugated goat anti-rabbit [F(ab’)2] (Thermo Fisher, Ref. A-11072; 1:500). Images were maximal intensity projections of serial Z-optical sections acquired using a Zeiss LSM 880 Airyscan microscope with a ×63 immersion objective (NA: 1.4), and pictures were analyzed with custom-made macros in Fiji software (38).

### ELISA

Levels of sgp130 were measured in serum samples of MS patients using commercial ELISA (R&D, Ref. DY228) following manufacturer’s instructions. Samples were diluted (1:100 and 1:200) and measured in duplicate. Absorbance was read in a Varioskan Flash (Thermo Fisher Scientific), and data were analyzed using GraphPad Prism v.7 software.

### Statistical Analysis

Data were analyzed with GraphPad Prism v.7 software and are presented as the mean ± standard error of the mean (SEM), unless otherwise indicated. Outliers were identified applying ROUT test and removed, and Shapiro–Wilk test was performed to determine if the variables were normally distributed. Mann–Whitney test, Wilcoxon signed-rank test, unpaired *t*-test, or paired *t*-test were performed to analyze the statistical difference between two groups. Kruskal–Wallis test, Friedman test (followed by Dunn’s multiple comparison test), or ANOVA test were used to compare differences between three or more groups. Correlation analyses were determined using Pearson correlation coefficient. Values of *p* ≤ 0.05 were considered significant (*), *p* ≤ 0.01 very significant (**), *p* ≤ 0.001 highly significant (***), and *p* ≤ 0.0001 extremely significant (****). For analysis of group-by-time interaction of the 2^-^Δ^ct^ values of *IL6ST*, *IL31RA*, and *SLC38A9* gene expression with those of *ANKRD55*, longitudinal data analysis was performed in moDC samples for four treatment conditions (immature control, immature AM580-treated, IFN-γ/LPS-matured control, and IFN-γ/LPS-matured AM580-treated) using generalized linear mixed univariable models. Random intercepts and unstructured variance-covariance matrix were used. Quantification of immunofluorescent images of ANKRD55 in the nucleus of moDC was performed as described before (39).

## Results

### Expression of *ANKRD55*, *IL6ST*, *IL31RA*, *DDX4*, *SLC38A9,* and Soluble gp130 Isoforms in Peripheral Blood Mononuclear Cell Subpopulations

MS risk alleles are associated with enhanced expression of *ANKRD55* in CD4^+^ T cells (30, 32), but genotypic effects on the expression of the flanking genes in PBMC subsets have not been reported. To identify cellular sources of transcripts in PBMC, five subpopulations were isolated from healthy donors: CD4^+^ T, CD8^+^ T, CD19^+^ B lymphocytes, CD14^+^ monocytes, and CD56^+^ NK cells. qPCR analysis was performed to assess mRNA levels of *ANKRD55* and neighboring genes including the isoforms sgp130, sgp130-RAPS, and sgp130-E10. Similar to *ANKRD55*, *IL6ST* was most highly expressed in CD4^+^ T-helper lymphocytes, but in contrast to it, it was also detectable in the other four subpopulations at a lower level of expression (**Supplementary Figure S2**). *IL31RA* was uniquely detected in CD14^+^ monocytes, and *DDX4* was undetectable in any of the PBMC subpopulations. *SLC38A9* was highly expressed in B lymphocytes but also detectable in the remaining subpopulations. The three soluble gp130 isoforms were highly expressed in CD4^+^ T helper lymphocytes but less so in the other subpopulations studied, similar to the patterns seen for *IL6ST* (**Supplementary Figure S2**). The influence of homozygosity for MS risk SNPs rs6859219 and rs7731626 and of the correlated SNP rs13186299 (explained below) on the expression in healthy control (HC) CD4^+^ T cells of *ANKRD55*, *IL6ST*, and the three sgp130 isoforms is shown in **Supplementary Figure S3**. Homozygotes for the risk alleles (CC, GG, and GG, respectively) expressed significantly higher levels of *ANKRD55* than those for the protective alleles, and directionally similar but weaker trends were seen for all other gene transcripts. In the non-CD4^+^ subsets, the rs13186299 G risk allele was significantly associated with lower *IL6ST* expression in CD56^+^ cells (*p* < 0.01) (**Supplementary Figure S4**).

### *ANKRD55* Is Expressed in Immature Monocyte-Derived Dendritic Cells and Induced by Retinoic Acid Receptor Alpha Agonist AM580

**Figure 2:**
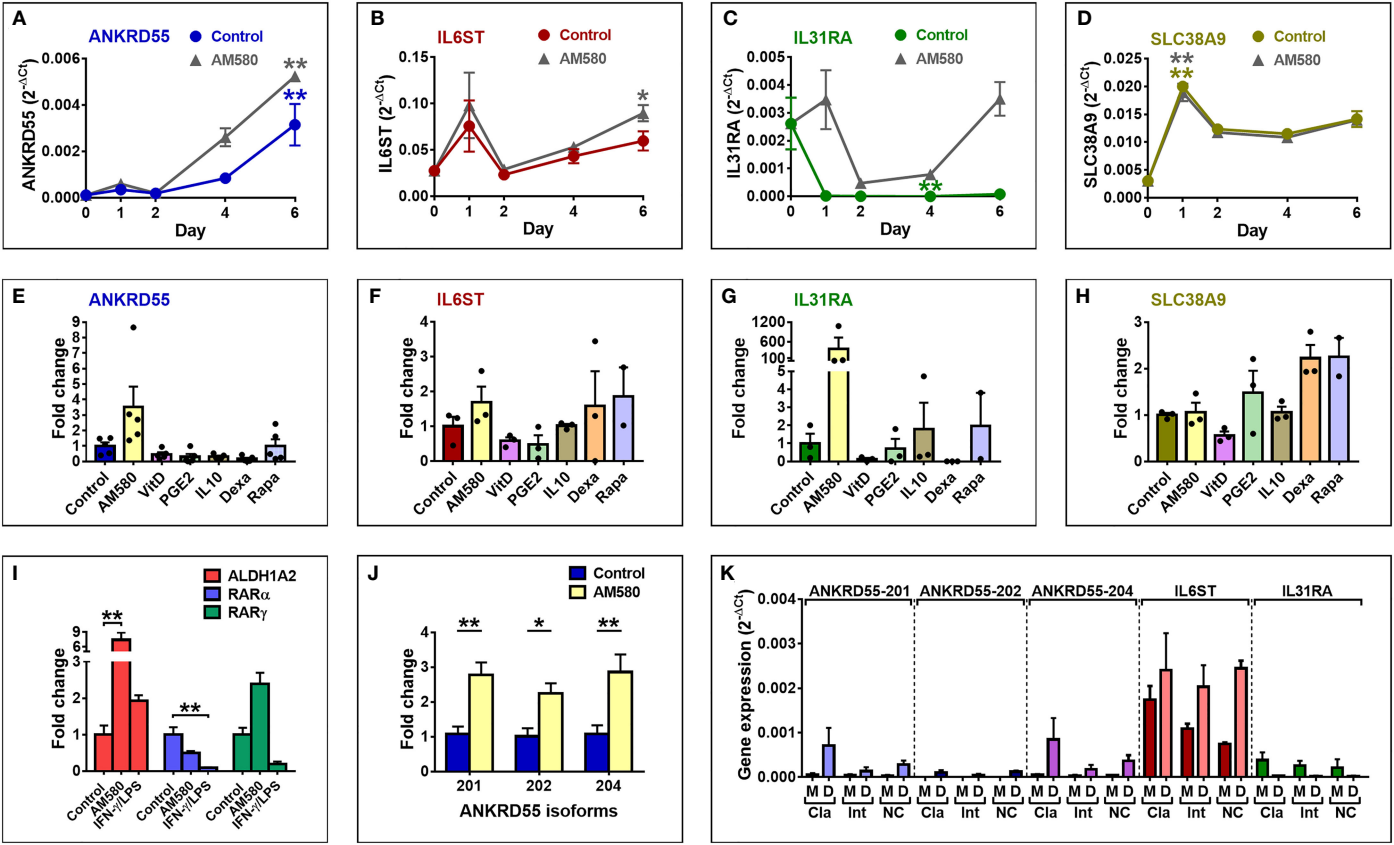
Expression of *ANKRD55* and neighboring genes in immature moDC. **(A–D)** Monocytes were cultivated for 6 days in IL-4/GM-CSF medium for differentiation into immature moDC in the absence (colored curves) or presence (gray curves) of AM-580, and expression levels of the indicated genes were measured by qPCR at the start of the cultivation (day 0) and on days 1, 2, 4, and 6. Mean ± SEM of 3 independent measurements; Friedman test (followed by Dunn’s multiple comparison test) for comparison of data points in each curve with day 0 and Wilcoxon test for comparison of both curves (Control vs. AM580). **(E–H)** Effect of tolerogenic compounds on gene expression in immature moDC by qPCR. Concentrations of compounds are provided in the *Materials and Methods*. Mean ± SEM of maximum 5 independent measurements; Friedman test (followed by Dunn’s multiple comparison test). **(I)** Analysis of ALDH1A2, RARα, and RARγ expression by qPCR. Mean ± SEM; *n* = 6; Friedman test (followed by Dunn’s multiple comparison test). **(J)** Expression levels of three discrete *ANKRD55* splice variants 201, 202, and 204 in immature moDC. Mean ± SEM; *n* = 10; Wilcoxon test. **(K)** Classical (Cla; CD14^hi^/CD16^-^), intermediate (Int; CD14^hi^/CD16^+^), and non-classical (NC; CD14^+^/CD16^+^) monocytes were isolated and separately cultivated for 6 days to differentiate into moDC. Gene expression levels were measured in the original monocyte subsets (M) and derived moDC (D). Mean ± SEM; *n* = 3; Mann–Whitney test for comparison between monocytes and moDC for each gene and subset. *p ≤ 0.05, **p ≤ 0.01.

We decided to study *ANKRD55* gene expression in monocyte-derived DC prompted by some earlier findings. Martin et al. (40) demonstrated, and we confirmed (37), high expression of the *IL22RA2* gene in immature moDC but not in monocytes or mature moDC. *IL22RA2* is located at an MS risk locus (3) and is poorly or not expressed in PBMC subsets. We hypothesized that its unique expression pattern perhaps indicates a role for immature moDC in MS pathogenesis, and that other MS risk genes may be expressed during the monocyte-to-DC (Mo–DC) differentiation stage. Thus, purified CD14^+^ monocytes from healthy donors were cultivated in Mo–DC differentiation medium containing IL-4 and GM-CSF for 6 days to induce formation of immature moDCs. Proper differentiation was assessed by CD209 (DC-SIGN) immunofluorescence (IF) microscopy and was associated with intense CD209 staining observed in >95% of cells. Cells were harvested for gene expression analysis by qPCR (**Figures 2A–D**) on days 0 (monocyte stage), 1, 2, 4, and 6 throughout this period. The *ANKRD55* and *IL6ST*, *IL31RA*, and *SLC38A9* genes were included in the analysis to assess differences in patterns of gene expression. *ANKRD55* gene expression started to rise from day 2 onward (*p* < 0.01 for comparison of levels on day 6 to those on day 0). In contrast, expression levels of *IL6ST* did not vary significantly, while those of *IL31RA* decreased (*p* < 0.01 for comparison of levels at day 4 vs. 0). *SLC38A9* expression levels peaked at day 1 (*p* < 0.01). Since expression of *DDX4* was not detected, this gene was not further studied. The effects of a series of tolerogenic compounds known to modulate gene expression in moDCs (41) including AM580, an analog of retinoic acid that acts as a selective retinoic acid receptor alpha (RARα) agonist, vitamin D3, IL-10, PGE2, dexamethasone, and rapamycin were tested by addition to Mo–DC medium over the course of the differentiation period. Of these, AM580 appeared to enhance mRNA levels both of *ANKRD55* (3.5-fold) and of *IL31RA* (379-fold), while the other compounds either inhibited the expression or had more modest effects on mRNA levels of any gene (**Figures 2E–H**). A qPCR time course analysis of the effect of AM580 on each of these genes compared to untreated cells was performed (gray curves in **Figures 2A–D**). In the presence of AM580, moDC produced slightly higher levels of *ANKRD55* and *IL6ST*. Since retinoic acid is essential for GM-CSF-induced *ALDH1A2* expression in DC (42), bioactivity of AM580 was ascertained through upregulation of *ALDH1A2* (*p* < 0.01 compared to untreated; **Figure 2I** **).** *RARA* and *RARG* were expressed in the moDC, with the former significantly affected (*p* < 0.01) only following treatment with IFN-γ/LPS.

Previously, we had found that CD4^+^ T lymphocytes coexpress three distinct *ANKRD55* mRNA splice variants, i.e., protein-coding isoforms *ANKRD55-201* (ENSEMBL nomenclature; full-length isoform; protein encoded Mr = 68.4 kDa) and *ANKRD55-202* (shorter protein-coding isoform; Mr = 36.9 kDa) and the non-coding processed transcript *ANKRD55-204*, which were originally designated isoforms 001, 005, and 007, respectively (30). To ascertain more robustly the effect of AM580 on *ANKRD55* expression in moDC, we performed qPCR using *ANKRD55* isoform-specific primer pairs each amplifying a unique *ANKRD55* splice variant, as described before (30). This analysis showed that immature moDCs express the three splice variants, and that AM580 significantly enhanced the expression levels of all three. Fold upregulation of transcripts 201, 202, and 204 by AM580 amounted to, respectively, 2.8 (*p* < 0.01 compared to untreated), 2.2 (*p* < 0.05), and 2.9-fold (*p* < 0.01) (**Figure 2J**).

Circulating monocytes can be distinguished based on relative expression levels of CD14 and CD16 surface proteins, namely, classical (CD14^hi^/CD16^-^), intermediate (CD14^hi^/CD16^+^), and non-classical (CD14^+^/CD16^+^) subsets. These are thought to contribute differentially to immune surveillance of the central nervous system (CNS) and to inflammatory responses in MS (43, 44). We assessed *ANKRD55* isoforms and *IL6ST* and *IL31RA* gene expression in these monocyte subsets separated by fluorescence-activated cell sorting (FACS) as well as in the corresponding immature DCs obtained by cultivation of each subset in Mo–DC differentiation medium for 6 days (**Figure 2K**). Very low but detectable levels of *ANKRD55* isoforms 201 and 204, though not of 202, were detected in all three monocyte subsets. The highest expression level was found in the classical subset, in line with Schmiedel et al. (45) who reported 0.4 transcripts per million (TPM) of *ANKRD55* in classical vs. 0.1 TPM in non-classical monocytes (45). Differentiation of the monocyte subsets into immature DC strongly enhanced expression levels of all three *ANKRD55* transcripts with highest levels for *ANKRD55-201* (11.5-fold upregulation) and *ANKRD55-204* (15.2-fold upregulation) found in the classical monocyte-derived DC subset. *IL6ST* was expressed at much higher levels than *ANKRD55* in the monocyte subsets but was less strongly increased in the moDC (1.4-fold upregulation in classical monocyte DC subset), while *IL31RA* was downregulated in each moDC subset.

### *ANKRD55* Expression Is Downregulated in IFN-γ/LPS-Matured Monocyte-Derived Dendritic Cells

**Figure 3:**
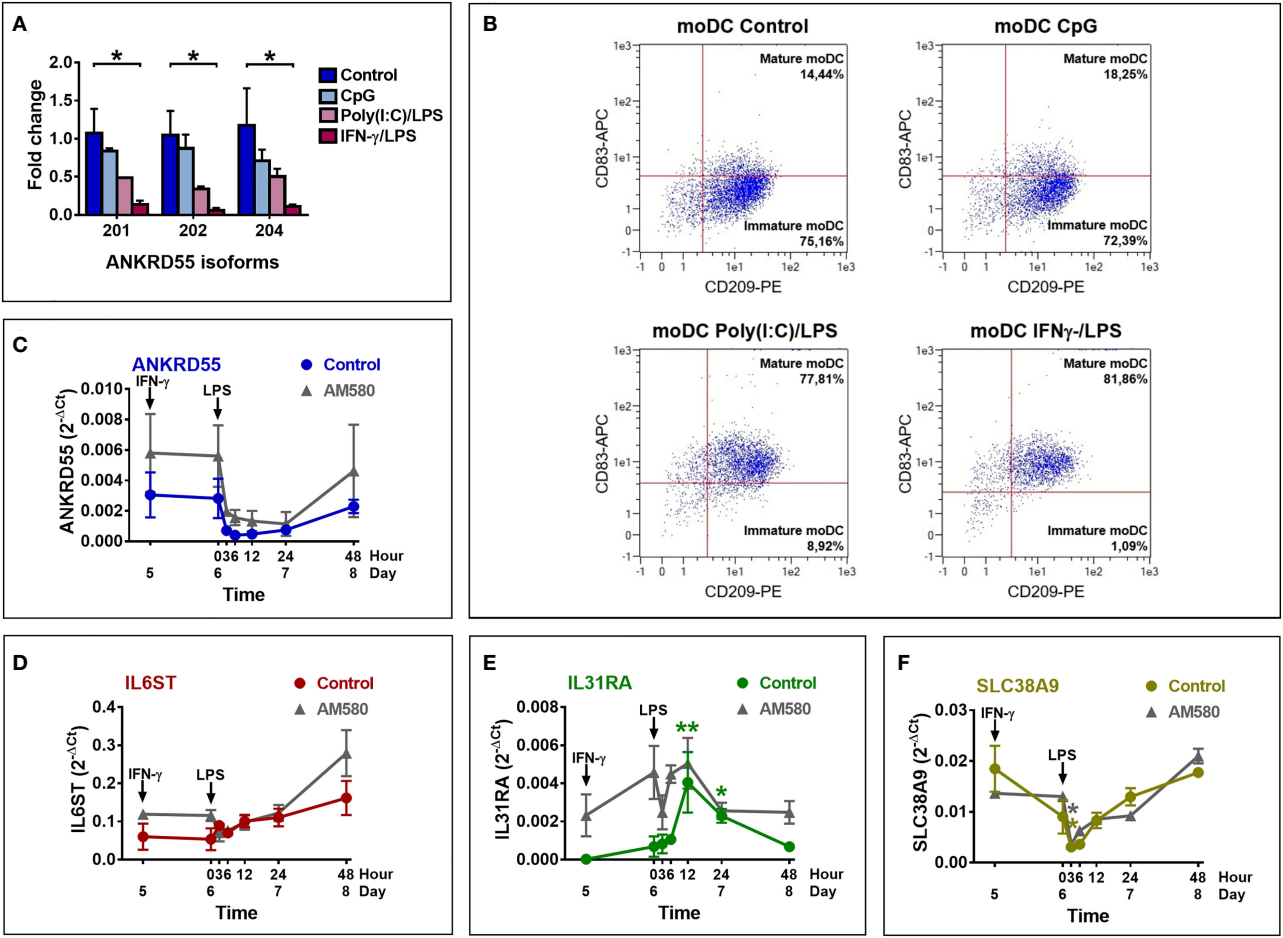
Effect of maturation of moDC on the expression of *ANKRD55*, *IL6ST*, *IL31RA*, and *SLC38A9*. **(A)** Comparison of the effect of maturation by CpG, poly(I:C)/LPS and IFN-γ/LPS on expression levels of the main *ANKRD55* splice variants by qPCR. Mean ± SEM; *n* = 3; Friedman test (followed by Dunn’s multiple comparison test). **(B)** Flow cytometry to assess the maturation percentage induced by the indicated stimuli based on the expression of CD209 (expressed in moDC) and CD83 (expressed in mature moDC only). Shown is a representative experiment out of 4 performed. **(C–F)** MoDCs cultivated for 5 days in MoDC medium were matured by addition of IFN-γ and 24 h later LPS. Gene expression was analyzed by qPCR coinciding with time of IFN-γ (day 5) and LPS (0 h) addition and 3, 6, 12, 24, and 48 h later. The experiment was also performed in cells differentiated in the presence of AM-580 (gray curves). Mean ± SEM of 3 independent measurements; Friedman test (followed by Dunn’s multiple comparison test) for comparison of data points in each curve with day 5 and Wilcoxon test for comparison of both curves (control vs. AM580). *p ≤ 0.05, **p ≤ 0.01.

We next assessed the effect of moDC maturation on the expression of *ANKRD55* and neighboring genes by treating the immature cells on day 6 for 6 h with Toll-like receptor (TLR)9 agonist CpG, or combinations of (TLR)4 agonist LPS and TLR3 agonist poly(I:C), or of IFN-γ and LPS (**Figure 3**). Percentage of mature cells assessed by flow cytometry of CD83 and CD209 (DC-SIGN) marker expression measured over 4 independent experiments was (average ± STDEV) 79.0% ± 8.9% with LPS + IFN-γ, 62.8% ± 12.4% with LPS + poly(I:C), and 23.75% ± 6.4% with CpG, while spontaneous maturation (control) amounted to 9.25% ± 5.1%. Expression levels of the three *ANKRD55* isoforms were measured and found to be significantly downregulated in moDC matured with IFN-γ/LPS (*p* < 0.05 vs. control; **Figures 3A, B** **)**, and similar weaker trends were seen for the other two conditions. In fact, extent of suppression of *ANKRD55* expression appeared proportional to the degree of moDC maturation. A time course analysis was performed to assess *ANKRD55* gene expression and that of its neighboring genes following initiation of maturation of moDCs with IFN-γ/LPS in the presence or absence of AM580. Maturation of moDC induced rapid downregulation of *ANKRD55* and *SLC38A9* (*p* < 0.05 at 3 h) gene expression with the lowest levels recorded from 3 to 12 h following addition of LPS (**Figure 3**), and this effect was seen in either untreated or AM580-pretreated cells. IFN-γ/LPS had little effect on *IL6ST* gene expression but induced high *IL31RA* expression levels in the untreated moDC (*p* < 0.01 at 12 h and *p* < 0.05 at 24 h after LPS addition for comparison with levels at day 5).

**Figure 4:**
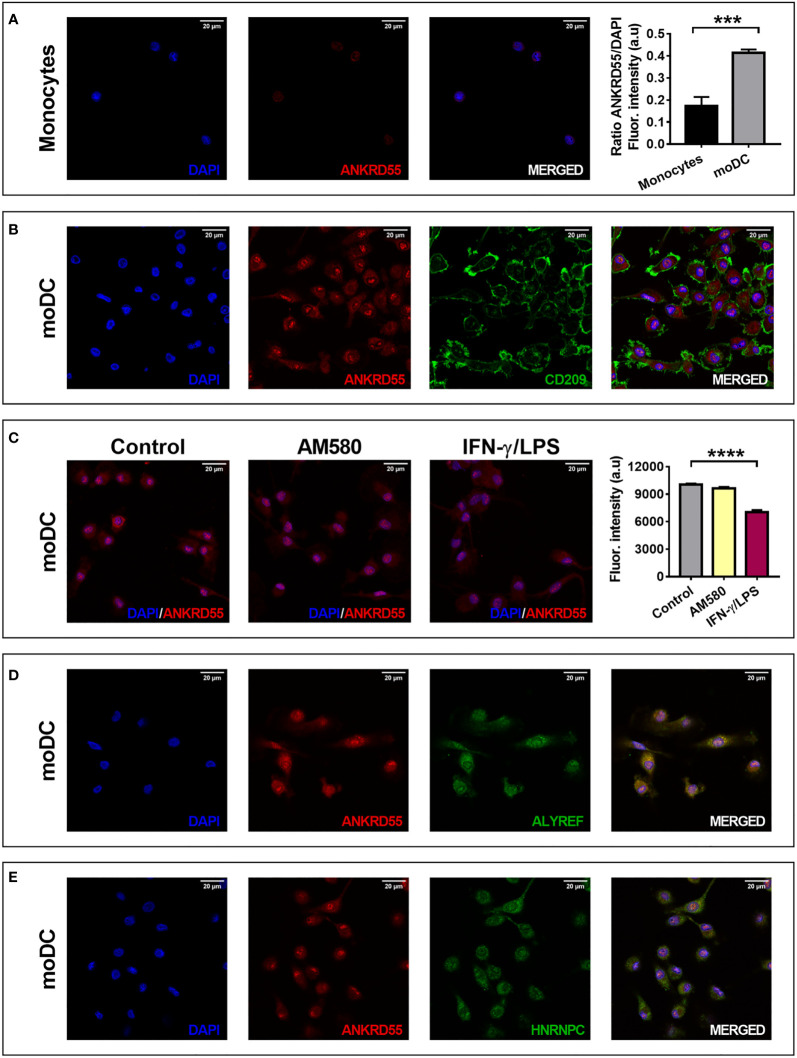
Localization of ANKRD55 in **(A)** monocytes (1.024 × 1.024 pixels = 0.131 microns/pixel) and **(B–E)** moDC by immunofluorescence microscopy. Rightmost graph represents mean ± SEM in monocytes and immature moDC (*n* = 5 cellular ROIs/group; unpaired *t*-test). **(B)** Colocalization of ANKRD55 with CD209, a membrane marker specific for moDC (2.048 × 2.048 pixels = 0.065 microns/pixel). **(C)** Effect of AM580 and IFN-γ/LPS treatment on nuclear ANKRD55 signal in immunofluorescence (1,808 × 1,808 pixels = 0,074 microns/pixel). The diagram on the right provides quantitative analysis of nuclear ANKRD55 immunofluorescence [mean ± SEM; *n* ≥ 59 cellular ROIs/condition; Kruskal–Wallis test (followed by Dunn’s multiple comparison test)]. **(D)** Colocalization of ANKRD55 with ALYREF (1.024 × 1.024 pixels = 0.131 microns/pixel). **(E)** Colocalization of ANKRD55 with HNRNPC (1.024 × 1.024 pixels = 0.131 microns/pixel). ***p ≤ 0.001, ****p ≤ 0.0001.

We used IF microscopy to detect intracellular location of ANKRD55. ANKRD55 was borderline visible in the nuclei of monocytes but stained clearly in the cytosol and in the nuclei of immature moDC. Differentiation of moDC was confirmed *via* costaining with CD209, a membrane marker that is absent in monocytes but appears strongly in moDC (**Figures 4A, B** **)**. Ratio nuclear ANKRD55/DAPI in moDC was significantly higher than that in monocytes (*p* < 0.001). We also compared immature, mature, and AM580-treated moDC. In a quantitative analysis of IF images, IFN-γ/LPS treatment significantly reduced nuclear ANKRD55 levels (*p* < 0.0001), while AM580 had no effect (**Figure 4C**). Given a nuclear pattern of irregular spots, we verified whether ANKRD55 could be associated with nuclear speckles, nuclear irregularly shaped dynamic structures (46). As shown in **Figures 4D, E**, ANKRD55 colocalized consistently with ALYREF and HNRNPC, two confirmed constituents of nuclear speckles (46).

**Figure 5:**
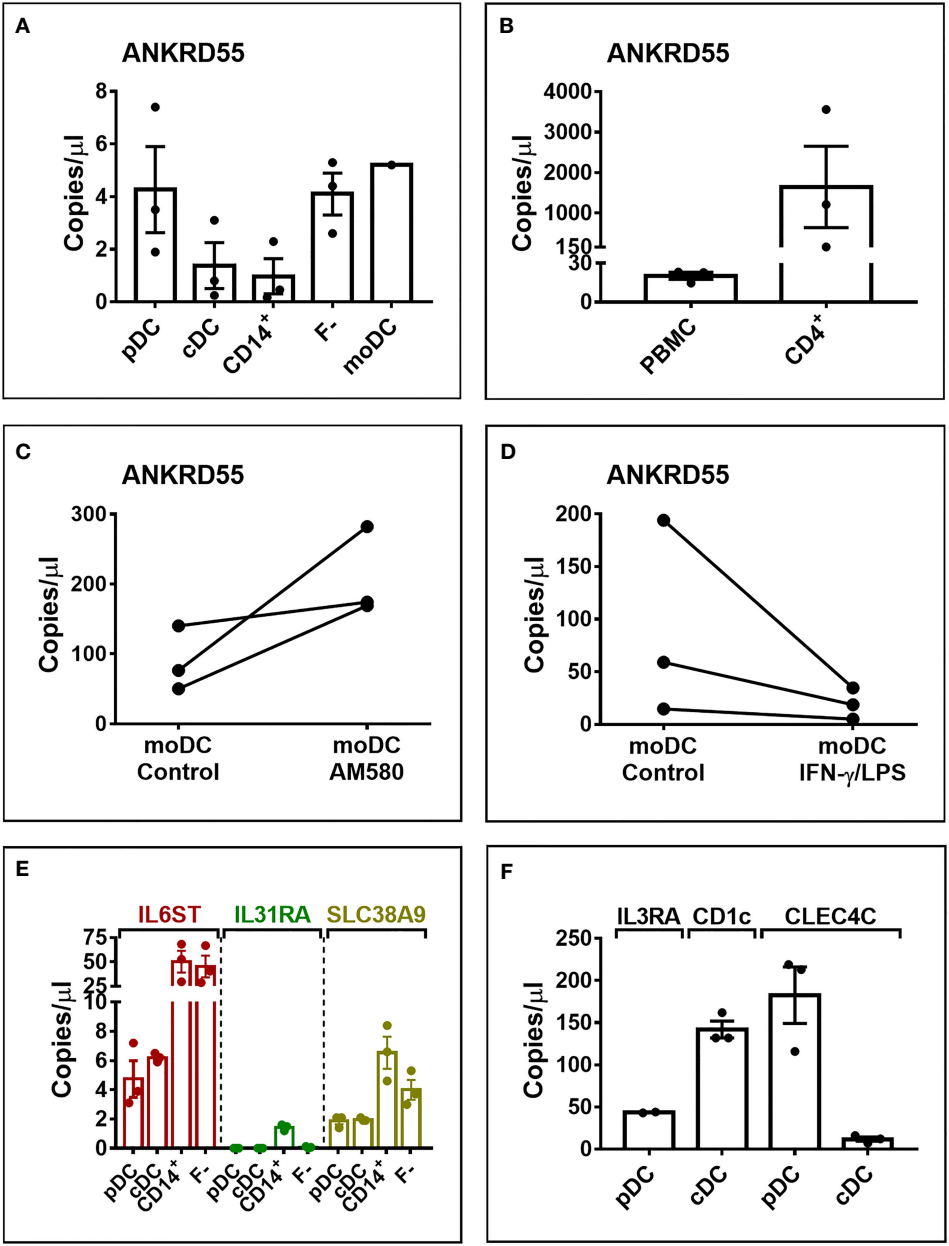
Digital droplet PCR (ddPCR) quantification of gene transcript copy numbers in pDC, mDC, CD14^+^ monocytes, PBMC fraction minus monocytes (F-), and moDC. Analysis was performed in 350 pg cDNA **(A, E, F)** or 30 ng of cDNA **(B–D)**. **(C)** ddPCR quantification of ANKRD55 transcript copy numbers in moDC treated with AM-580. **(D)** *ANKRD55* copy numbers in moDC matured or not with IFN-γ/LPS measured at 6 h after addition of stimulus. **(F)** Transcript levels of markers specific for mDC (CD1c) and pDC (IL3RA, CLEC4C).

We further performed absolute quantification by ddPCR of transcript levels in natural circulating conventional (cDC) and plasmacytoid (pDC) dendritic cells, CD14^+^, and CD4^+^ cells, and in untreated and treated moDC (**Figure 5**). As measured in 350 pg cDNA, *ANKRD55* transcript copy numbers per μl (average ± STDEV) in pDC (4.3 ± 2.8) and mDC (1.4 ± 1.5) were slightly higher than those in CD14^+^ monocytes (1 ± 1.15) (**Figure 5A**). In contrast, *IL6ST*, *IL31RA*, and *SLC38A9* were expressed at much higher levels in CD14^+^ cells than those in pDC or mDC (**Figure 5E**). AM580 enhanced basal *ANKRD55* transcript levels in immature moDC at day 6 from 88.8 ± 46.2 to 208.3 ± 63.8 copies/μl, and IFN-γ/LPS downregulated day-5 immature moDC *ANKRD55* levels from 89.3 ± 93.3 to 19.5 ± 14.9 copies/μl, both measured in 30 ng of cDNA (**Figures 5C, D** **)**. In comparison, CD4^+^ T cells expressed much higher levels of *ANKRD55* (1,643 ± 1,741 copies/μl) also measured in 30 ng cDNA (**Figure 5B**).

A longitudinal data analysis was performed in order to determine whether the curves representing *ANKRD55* and *IL6ST*, *IL31RA*, and *SLC38A9* mRNA expression levels (**Figures 2**, **3**) are statistically dissimilar within each treatment condition. The *p*-values corresponding to the group-by-time interaction term’s significance in the adjusted generalized linear mixed univariable models are collected in **Table 1**. The results show that *IL31RA* gene expression showed significant group-by-time interactions with *ANKRD55* in all tested conditions. Group-by-time interactions, however, were not statistically significant when comparing *ANKRD55* with *IL6ST* or with *SLC38A9* in both control and AM580-administered groups of the immature sample and with *SLC39A9* in both control and AM580-administered groups of the mature sample. Therefore, in those cases, the similarity between the analyzed groups in terms of the trend among the evaluated times cannot be rejected. Subsequent genetic analysis was performed focusing on *ANKRD55* and *IL6ST* in the immature untreated moDC.

**Table 1:** *p*-Values corresponding to the group-by-time interaction term’s significance in the adjusted generalized linear mixed univariable models.

Compared evolutions	Immature		Mature	
	Control	AM580	Control	AM580
*ANKRD55* vs. *IL6ST*	0.5772	0.2973	0.0016	0.0142
*ANKRD55* vs. *IL31RA*	0.0001	0.0010	0.0304	0.0005
*ANKRD55* vs. *SLC38A9*	0.7150	0.8674	0.4231	0.0972

### Multiple Sclerosis Risk Single-Nucleotide Polymorphisms Modulate the Expression of *ANKRD55* and *IL6ST* in Immature Monocyte-Derived Dendritic Cells

**Figure 6:**
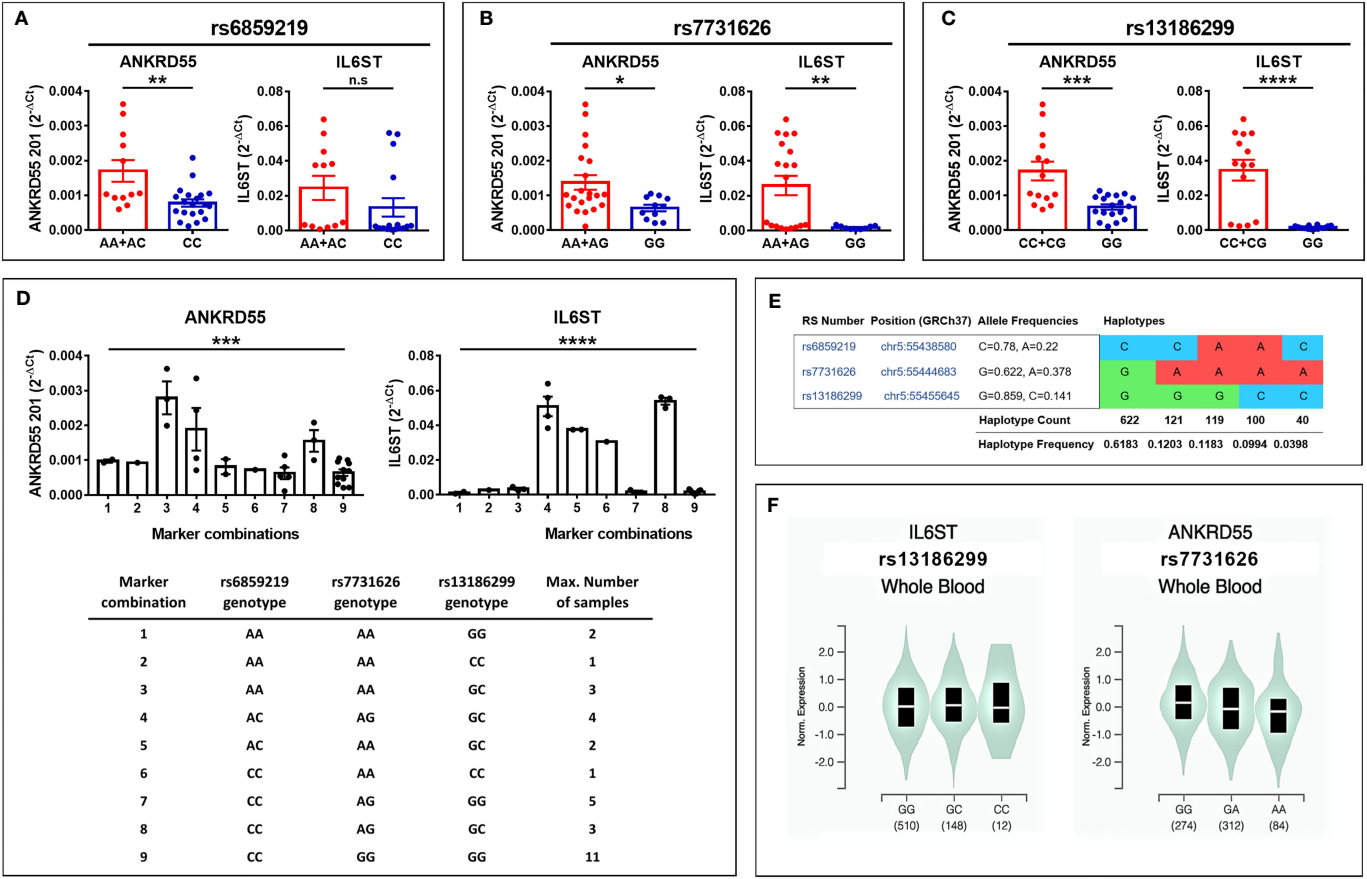
**(A–C)** Modulation of *ANKRD55* and *IL6ST* gene expression in immature moDC of healthy donors by *cis* genomic MS risk variants (mean ± SEM; *n* ≥ 28; Mann–Whitney test); **(A)** rs6859219, **(B)** rs7731626, and **(C)** the correlated *ANKRD55*-intronic SNP rs13186299. **(D)** Assessment of *ANKRD55* and *IL6ST* expression levels according to three-SNP marker combinations (mean ± SEM; *n* ≥ 28; one-way ANOVA test). **(E)** Haplotype distribution in the European population of the three SNPs. Image information was produced by means of ldlink.nci.nih.gov. **(F)** Genomic variants rs13186299 (left) and rs7731626 (right) are the most significant single-tissue eQTLs for *IL6ST* and *ANKRD55* in whole blood, respectively, based on information collected from the GTEx portal (gtexportal.org/home/). The risk genotype of each of both SNPs is GG, and the protective allele is C for rs13186299 and A for rs7731626. *p ≤ 0.05, **p ≤ 0.01, ***p ≤ 0.001, ****p ≤ 0.0001. n.s., not significant.

To assess whether *ANKRD55* MS risk genotypes regulate *ANKRD55* and *IL6ST* expression in immature moDCs, we performed qPCR in day-6 immature moDCs generated from healthy subjects who were genotyped for MS risk SNPs rs6859219 and rs7731626. Compared to the risk alleles, the protective A alleles of both SNPs were associated with higher mRNA expression of *ANKRD55* (*p* < 0.01 and *p* < 0.05, respectively) and of *IL6ST* (not significant trend and *p* < 0.01, respectively) (**Figures 6A, B** **)**. The mRNA levels of sgp130 and sgp130-RAPS in immature untreated moDC appeared, as well, to be higher in carriers of the A alleles (**Supplementary Figure S5**). Notably, in CD4^+^ T lymphocytes, the opposite directional effect is observed with the A alleles associated with lower levels of *ANKRD55* compared to the risk alleles (27, 28, 31; also, in **Supplementary Figure S3**). The opposite effects in moDC could be due to linkage disequilibrium (LD) with distinct genomic variants correlated to specific phenotypic traits of myeloid or monocytic cells. We performed a search for GWAS variants in LD with these SNPs and identified two candidate SNPs meeting these criteria, rs28722705 (Chr 5: 56158115) and its proxy rs13186299 (Chr 5: 56159818) (*D’* = 1; *R^2^* = 1*)*, which emerged with genome-wide significance from GWAS on monocyte count (*p* ≤ 5 × 10^-27^; 5, 6) and monocyte percentage of white cells (*p* = 7 × 10^-22^; 7), respectively. Both are intron variants located in *ANKRD55* (**Figure 1**) that occur in partial LD with both *ANKRD55* MS risk SNPs (*D’* = 0.62–0.98; *R^2^* = 0.22–0.26). Interestingly, in the PheWAS database (that integrates studies by the FinnGen, UK Biobank, and GWAS Catalog consortia; genetics.opentargets.org), MS risk SNPs rs7731626 and rs6859219 are reported to be associated with monocyte count with genome-wide significance (*p* = 1.8 × 10^-14^ and *p* = 5.2 × 10^-19^, respectively), suggesting colocalization of MS risk and monocyte count traits. The Variant-to-Gene pipeline (genetics.opentargets.org) that integrates QTL data, Promoter Capture Hi-C, *in silico* functional predictions, and variant distance to gene prioritizes *ANKRD55* and *IL6ST* as the genes functionally implicated by all four variants. In the GTEx portal, rs13186299 is recorded as the most significant single-tissue eQTL for *IL6ST* expression in whole blood (*p* = 4.4 × 10^-11^; normalized effect size 0.17) (**Figure 6F**).

Reanalysis of the expression data in the sample cohort now genotyped for rs13186299 (**Figures 6C, D** **)** revealed a highly significant association of mRNA levels of *ANKRD55* and *IL6ST* in immature moDC with genotype (*p* < 0.001 and *p* < 0.0001, respectively), identifying this SNP as exerting a higher impact upon *ANKRD55* and *IL6ST* expression in moDC than either MS risk SNP. Expression levels showed roughly similar variations according to marker combination (exception made for combination 3), with the rs13186299 GG genotype always segregating with low levels of either gene product. In the European population, the MS risk SNP rs7731626 G allele is uniquely found in a single haplotype together with the rs13186299 G allele (**Figure 6E**). Therefore, homozygotes for the rs7731626 G allele, corresponding to 38% of European rs7731626 sample genotypes, are predicted to display genetically determined low levels of both genes in moDC. Levels of *ANKRD55* splice variants induced by AM580 in immature moDC compared to untreated cells were significantly higher in homozygous carriers of MS risk alleles but not significantly different in homozygotes of the protective alleles, though the scarcity of the latter genotype restricted the power of the test in this group (**Supplementary Table S3**).

### *ANKRD55* and *IL6ST* Gene Expression Patterns in CD4^+^ T Cells and Monocyte-Derived Dendritic Cells of Multiple Sclerosis Patients

**Figure 7:**
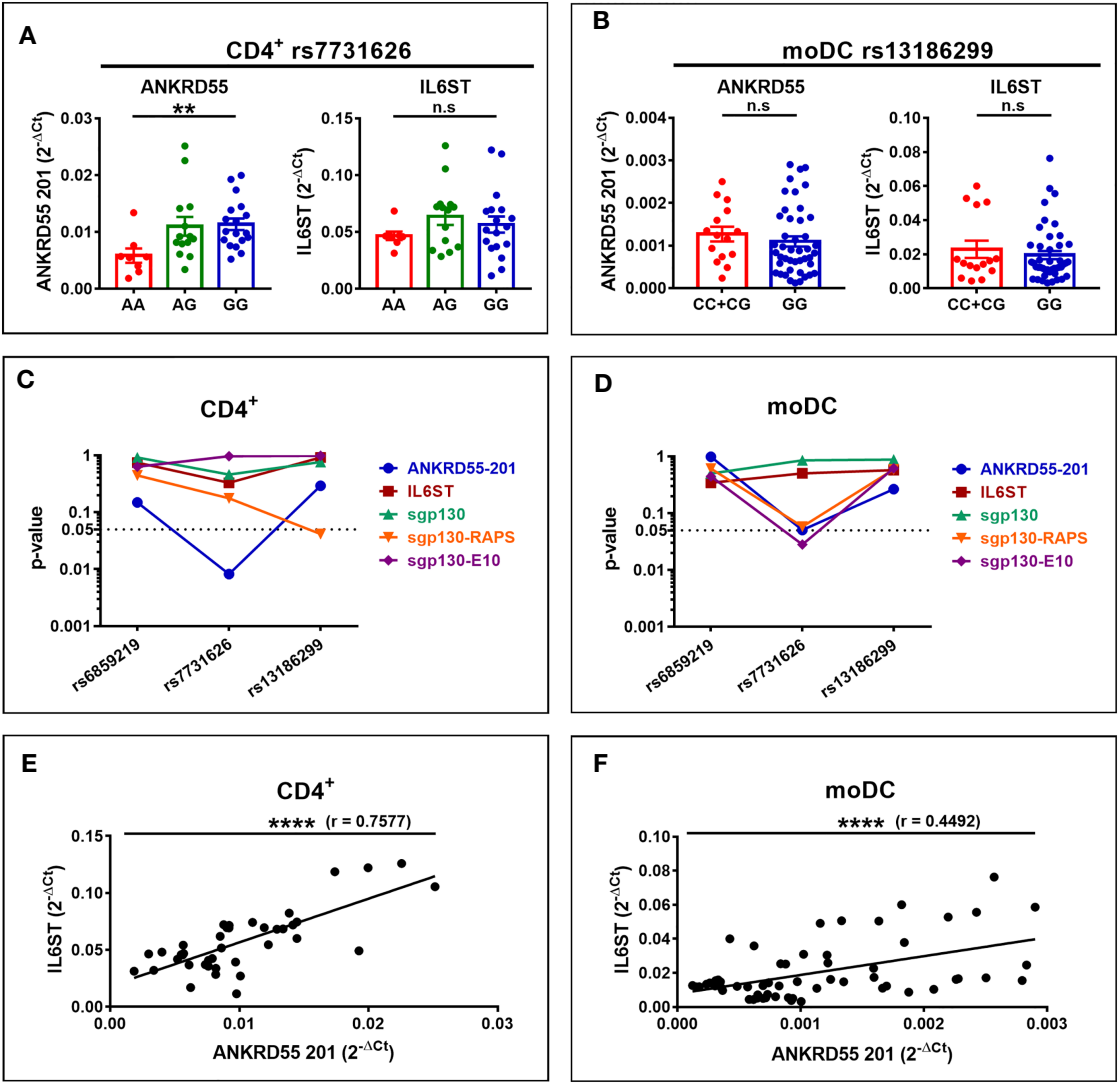
Expression of *ANKRD55*, *IL6ST*, and sgp130 isoforms in CD4^+^ T cells **(A, C)** and immature moDC **(B, D)** of MS patients. Panels **(A, B)** represent the effects of rs7731626 genotype in CD4^+^ T cells (mean ± SEM; *n* = 40, Cohort 2; Kruskal–Wallis test) and of rs13186299 genotype in moDC (mean ± SEM; *n* = 59, Cohorts 1 and 2; Mann–Whitney test), respectively. **(C, D)** Plot *p*-values for the three SNPs’ effects on the expression of *ANKRD55*, *IL6ST*, sgp130, sgp130-RAPS, and sgp130-E10 in CD4^+^ T cells and moDC (Mann–Whitney or Kruskal–Wallis tests). **(E**, **F)** represent Pearson correlation of *ANKRD55* and IL6ST 2^-Δct^ values in CD4^+^ T cells and in moDC of MS patients from panels **(A, B)**, respectively. **p ≤ 0.01, ****p ≤ 0.0001. n.s., not significant.

**Figure 8:**
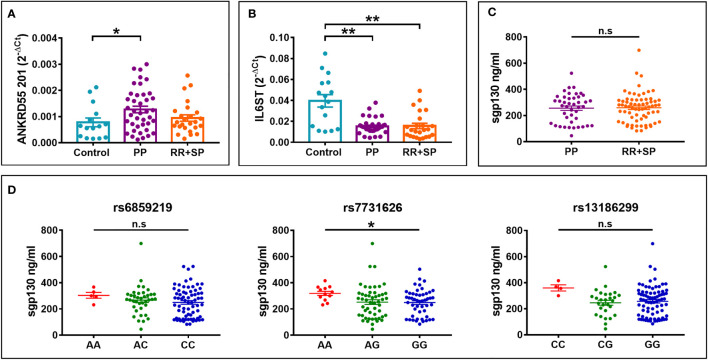
Differential **(A)** *ANKRD55* and **(B)** *IL6ST* gene expression levels in immature moDC according to clinical course of MS, PP, or RR+SP, as measured by qPCR. Mean ± SEM; *n* = 80, Cohorts 1 and 2, Kruskal–Wallis test (followed by Dunn’s multiple comparison test). **(C)** Levels of serum sgp130 protein measured by ELISA in PP MS and RR+SP MS. Mean ± SEM; *n* = 112, Cohorts 1, 2, and 3, Mann–Whitney test (n.s.). **(D)** Serum sgp130 levels in MS patients from panel **(C)** stratified for rs6859219, rs7731626, or rs13186299 genotype. Kruskal–Wallis test (followed by Dunn’s multiple comparison test). *p ≤ 0.05, **p ≤ 0.01. n.s., not significant.

We next set out to verify whether these genetic influences on *ANKRD55* and *IL6ST* gene expression are measurable in CD4^+^ T lymphocytes and immature moDCs derived from CD14^+^ monocytes freshly isolated from venous blood samples from untreated MS patients (clinical and demographic data of MS patients in **Supplementary Table S1**). In CD4^+^ T cells, rs7731626 genotype was significantly associated with mRNA expression levels of *ANKRD55* (*p* < 0.01) but not with that of *IL6ST*, while the other two SNPs were not associated with expression levels of any gene (**Figures 7A, C** **)**. In immature moDCs, no association between genotype of any SNP and expression level of *ANKRD55* or *IL6ST* was observed (**Figures 7B, D** **)**. It is worth noting that expression levels of both genes in moDC of rs13186299 GG homozygotes were higher in MS patients (**Figure 7B**) than in healthy controls (**Figure 6C**) (trending at *p* = 0.12 for *ANKRD55* and significant for *IL6ST* at *p* < 0.0001 upon comparison of both groups), while those of C carriers were not significantly different (*p* > 0.05 for comparison of both groups). This observation is suggestive for allele-restricted preconditioning of CD14^+^ monocytes in MS patients. Expression levels of the three sgp130 isoforms, i.e., sgp130, sgp130-RAPS, and sgp130-E10, were also measured by qPCR but did not vary with SNP genotype, though two weaker associations (*p* < 0.05; CD4^+^ sgp130-RAPS for rs13186299 and moDC sgp130-E10 for rs7731626) were observed (**Figures 7C, D** **)**. We calculated Pearson correlation coefficients between individual *ANKRD55* and *IL6ST* mRNA expression levels in either cell subset. In immature moDC, expression levels of *IL6ST* and *ANKRD55* were moderately positively correlated with Pearson’s r = 0.45 (*p* < 0.0001), while in CD4^+^ T cells, their expression levels were more strongly correlated with Pearson’s r = 0.76 (*p* < 0.0001) (**Figures 7E, F** **)**. We considered clinical course as a variable. In moDC, increased *ANKRD55* gene expression was observed in PP MS patients compared to HC (*p* < 0.05), as well as decreased *IL6ST* expression in both PP MS and RR+SP MS groups (*p* < 0.01; **Figures 8A, B** **)**. Finally, we quantified serum levels of sgp130 by ELISA in MS patients. Sgp130 levels did not differ in PP vs. RR+SP patients (**Figure 8C**) but showed significant variation according to rs7731626 genotype. Higher levels of sgp130 were observed in homozygotes of the protective allele (*p* < 0.05), and similar trends were seen with both other SNPs (**Figure 8D**).

## Discussion

In the present study, we demonstrate that the *ANKRD55* gene is induced in monocytes during differentiation into immature moDC in the presence of IL-4/GM-CSF. Expression of *ANKRD55* in these cells is enhanced by a retinoic acid agonist and suppressed by maturation with IFN-γ/LPS treatment, while expression of *IL6ST*, the adjacent gene, appears less affected by either of these treatments. Expression levels of both genes in immature moDC are similarly modulated by the MS risk SNPs rs6859219 and rs7731626, with opposite directional effect to that seen in T lymphocytes: MS risk alleles are associated with higher levels of *ANKRD55* and *IL6ST* in CD4^+^ T lymphocytes of healthy controls (29–32) but, as shown here, with lower levels in immature moDC compared to the protective alleles. Though we did not perform a formal eQTL study, further analysis revealed that these allelic effects in moDC are arguably due to LD of the MS risk SNPs with another nearby intronic SNP in *ANKRD55*, rs13186299. This SNP has not arisen from GWAS in MS but from a GWAS on monocyte percentage of white blood cells (7) and is the main GTEx eQTL SNP for *IL6ST* expression in whole blood. Tissue-specific opposite eQTL effects have been observed for GWAS SNPs associated with complex traits including MS, with a fraction of these showing discordant effects in untreated CD4^+^ T cells and monocytes (45, 47, 48). Around 30% of the 200 GWAS autosomal risk variants contained within the current MS genomic map can be tagged to *cis*-QTL effects on gene expression in either naive CD4^+^ T cells or monocytes (3). Five SNPs passed the FDR threshold for expression of *ANKRD55* in CD4^+^ T cells and PBMCs but not in monocytes or brain (3). Our work now links *ANKRD55* MS risk SNPs already associated with a constitutive regulatory *cis* effect on *ANKRD55* and *IL6ST* gene expression in CD4^+^ T cells now to a distinct regulatory element mediating an opposite IL-4/GM-CSF-induced *cis* effect in monocytes.

MoDCs, used widely as an *in vitro* DC model, are in fact a highly plastic cell type capable of acquiring a multitude of discrete functions. They are closely related to and share transcriptomic gene signatures with naturally occurring inflammatory DCs that emerge from monocytes under inflammatory conditions (49–52) and that do not develop *via* a common DC precursor in contrast to pDC and cDC. Inflammatory DCs show considerable phenotypic heterogeneity, and they function mainly at a site of inflammation rather than migrating to lymph nodes. One such subset, SLAN^+^ DC, represents a subfraction of CD14^+^/CD16^+^ monocytes with pronounced pro-inflammatory activities that has been shown to accumulate in highly inflammatory brain lesions in MS patients (53). More recently, single-cell RNA sequencing studies identified the DC subset DC4 that is marked by high CD16, shares signatures with monocytes, and may be related to SLAN^+^ cells (51, 52). Though their role in MS is still poorly understood, inflammatory DCs are known to stimulate CD4^+^ T cells to produce IL-17 and induce Th17 differentiation from naive CD4^+^ T cells (49). These activities may be mediated through IL-6 trans-signaling into T cells that abrogates *de novo* induction of autoimmune inhibitory regulatory T cells (Tregs) (54) and promotes differentiation of pathogenic Th17 cells (55). Th17 cells are crucial contributors to CNS inflammation in MS (56). These effects can be modulated by sgp130, which inhibits IL-6 trans-signaling by binding to the IL-6/sIL-6R complex, thus blocking its interaction with gp130 cell surface receptors (57).

Given this context, as part of this study, we analyzed the expression of three secreted gp130 isoforms (sgp130, sgp130-RAPS, and sgp130-E10) produced by the *IL6ST* gene by qPCR and of circulating sgp130 by ELISA. In CD4^+^ T cells of HC, MS risk alleles when compared to protective alleles are associated with higher sgp130 isoform mRNA levels, while in moDC, they are associated with lower levels. Serum levels of sgp130 were higher in MS patients, homozygote of the protective allele of MS risk SNPs, reaching significance for rs7731626 (**Figure 8D**), and this is concordant with a recent publication showing significantly increased serum sgp130 levels in MS carriers of the protective allele of the correlated MS risk SNP rs71624119 (58; **Figure 1**). In the latter study, during fingolimod treatment, serum levels of sgp130 increased in all patients but to a higher degree in patients who are homozygous for the risk allele (58). Intrinsically, findings from our study and the study by Bedri et al. (58) are compatible with the trans-signaling model of higher protection against MS risk through increased sgp130-mediated downregulation of IL-6/sIL6R-dependent pro-inflammatory activities and highlight these MS risk SNPs as potential determinants of circulating sgp130. However, this pattern is incongruent with the rs7731626 genotype effect on sgp130 mRNA levels in CD4^+^ T lymphocytes (**Supplementary Figure S3**), which shows a pattern in homozygotes that is opposed to that of serum sgp130 levels (**Figure 8D**). One possible explanation is that rs7731626, probably *via* LD with eQTL-linked genomic variants, such as rs13186299, may affect serum sgp130 production by other PBMC subsets in opposite allelic direction to that in CD4^+^ T lymphocytes. DICE database^2^ indicates opposite genotype effects for rs7731626 on *IL6ST* transcription in B cells, classical and non-classical monocytes, and NK cells when compared to CD4^+^ T cells. Still, among PBMC, CD4^+^ T lymphocytes, the most abundant PBMC subset, are comparatively also the highest producers of *IL6ST* and sgp130 mRNA (Protein Atlas; **Supplementary Figure S2**). The genotype-serum sgp130 ELISA data in this study that contrasts with this could refer therefore in fact to a relatively limited contribution of CD4^+^ T lymphocytes to circulating levels of sgp130 or to additional sgp130-producer contributors from vessel walls or non-PBMC subsets. It may also point to as yet undisclosed effects of genomic *cis*-variants affecting cell-specific sgp130 isoform splicing or stability, and/or posttranslational processing and trafficking of sgp130, that are correlated to the main MS risk SNP. A missense variant (Gly148Arg) in exon 5 of the IL6ST gene, rs2228044, that has been associated with higher serum sgp130 levels (59) is not in LD with rs7731626 (*R^2^* = 0.0008 in the European population), ruling out a mechanistic correlation. In light of these current observations, and considering serum sgp130 appears to be a polygenic trait influenced by genomic variants acting in *trans* (60), decreased circulating sgp130 levels constitute likely a secondary effect of the major causative link to MS risk conferred by rs7731626, which thus far is understood as upregulation of both *ANKRD55* and *IL6ST* expression in CD4^+^ T lymphocytes (34, 36).

Though the expression of both these genes has been reported to be affected to a similar extent in HC by rs7731626 (34), we show that in MS patients, rs7731626 is significantly associated with *ANKRD55* expression in the CD4^+^ T cells of MS patients (**Figure 7A**). Roostaei et al. (35) reported a *cis*-eQTL effect for this SNP on *ANKRD55* expression in both naive and memory CD4^+^ T cells of MS patients. In immature moDC of MS patients, *ANKRD55* and *IL6ST* expression was independent of the tested SNPs, in contrast to HC, whose expression levels of either gene were significantly associated with rs7731626 and rs13186299. This discrepancy may be the result of preconditioning of blood monocytes by factors enriched in serum of MS patients, prior to their isolation and differentiation into moDC, and was only seen in rs13186299 GG homozygotes. Though we did not perform a functional analysis, we assessed whether clinical course could be a priming factor affecting moDC expression of both genes. In the PP form of MS, but not in RR or SP MS, blood monocytes are characterized by an IL-1β signature consequential to NLRP3 inflammasome overactivation (61). Analysis of immature moDC generated from circulating monocytes of PP, RR, and SP MS patients revealed enhanced expression of *ANKRD55* uniquely in the PP MS group, while *IL6ST* was decreased in all MS patients regardless of clinical course. These effects were independent of genotype, which therefore in HC but less so in MS determines moDC coregulation of *ANKRD55* and *IL6ST*.

In this study, ANKRD55 was observed in both cytosolic and nuclear compartments of moDC. We found that nuclear ANKRD55 colocalized with proteins typically contained within nuclear speckles. Nuclear speckles were originally thought to be sites for storage and modification of RNA splicing factors but are now recognized as hubs for integrating all the nuclear gene expression regulation steps (46). Previously, using a distinct antibody to ANKRD55 and different permeabilization methods (27), we could not demonstrate clearly its nuclear localization in HEK293 cells by IF but demonstrated its association with the mitotic spindle. However, the enrichment of RNA-binding proteins in the HEK293 nuclear interactome of ANKRD55 (27) is compatible with a nuclear speckles-based location seen here, as some proteins we identified in that interactome are established NS proteins including the RNA helicases DDX3X, DDX21, nuclear RNA export factor NXF1, RNA-binding protein RBM14, and RNA stabilizer ELAVL1 (27, 46).

In conclusion, this study reports coregulation of *ANKRD55* and *IL6ST* in immature moDCs by genomic variants associated with MS risk. Though little is known about ANKRD55’s biological relevance to autoimmunity, its expression in immature moDC and upregulation by retinoic acid and downregulation by TLR maturation factors suggest a specific role associated with the differentiation stage of monocyte precursor to DCs. Our study highlights the importance of performing more extensive genome-wide QTL studies in intermediate or induced states of immune cells in order to gather an integrated understanding of the multifarious expression of MS risk conferred by associated genomic variants.

## Data Availability Statement

The original contributions presented in the study are included in the article/**Supplementary Material**. Further inquiries can be directed to the corresponding author.

## Ethics Statement

The studies involving human participants were reviewed and approved by the Comité Ético de Investigación Clínica de Euskadi, the Comité Ético de Investigación Clínica Hospital Universitari Vall D’Hebron, and the Comité Ético de la Investigación del Hospital Ramón y Cajal. The patients/participants provided their written informed consent to participate in this study.

## Author Contributions

JM, IA, RTN, AAl, JDG, AVE, SM, and NV contributed to the acquisition and analysis of the experimental data. CL provided assistance and antibodies for immunofluorescence microscopy. AAn, SB, MM-B, AAA, JLSM, LM, XM, LMV, and MC provided biological samples and clinical data of MS patients. The article was written by JLSM and KV. All authors edited the article. All authors contributed to the article and approved the submitted version.

## Funding

This research was supported by grants to KV from MINECO (SAF2016-74891-R), Instituto de Salud Carlos III (FIS-PI20/00123), Gobierno Vasco RIS3 (Ref. 2019222043), and Red Española de Esclerosis Múltiple (REEM; RD16/0015/0005). NV and LMV were supported by ISCIII (FIS-PI18/00572) and REEM (RD16/0015/0001). RTN is a recipient of a fellowship from the Secretaría Nacional de Ciencia y Tecnología e Innovación (SENACYT; Convocatoria Doctorado de Investigación Ronda III, 2018; Ref. BIDP-III-2018-12) of the Gobierno Nacional, República de Panamá.

## Conflict of Interest

The authors declare that the research was conducted in the absence of any commercial or financial relationships that could be construed as a potential conflict of interest.

## Publisher’s Note

All claims expressed in this article are solely those of the authors and do not necessarily represent those of their affiliated organizations, or those of the publisher, the editors and the reviewers. Any product that may be evaluated in this article, or claim that may be made by its manufacturer, is not guaranteed or endorsed by the publisher.

## Footnotes

1 https://www.gtexportal.org/home/gene/ANKRD55

2 https://dice-database.org
