# Supplementary material for: Genomic Multiple Sclerosis Risk Variants Modulate the Expression of the ANKRD55–IL6ST Gene Region in Immature Dendritic Cells
Source: Front Immunol. 2022 Jan 17;12:816930. doi: 10.3389/fimmu.2021.816930 (PMC8801523; doi:10.3389/fimmu.2021.816930)
Supplement: Supplementary file 1 [file DataSheet_1.docx]

Supplementary Material

**Genomic multiple sclerosis risk variants modulate expression of the ANKRD55 – IL6ST gene region in immature dendritic cells**


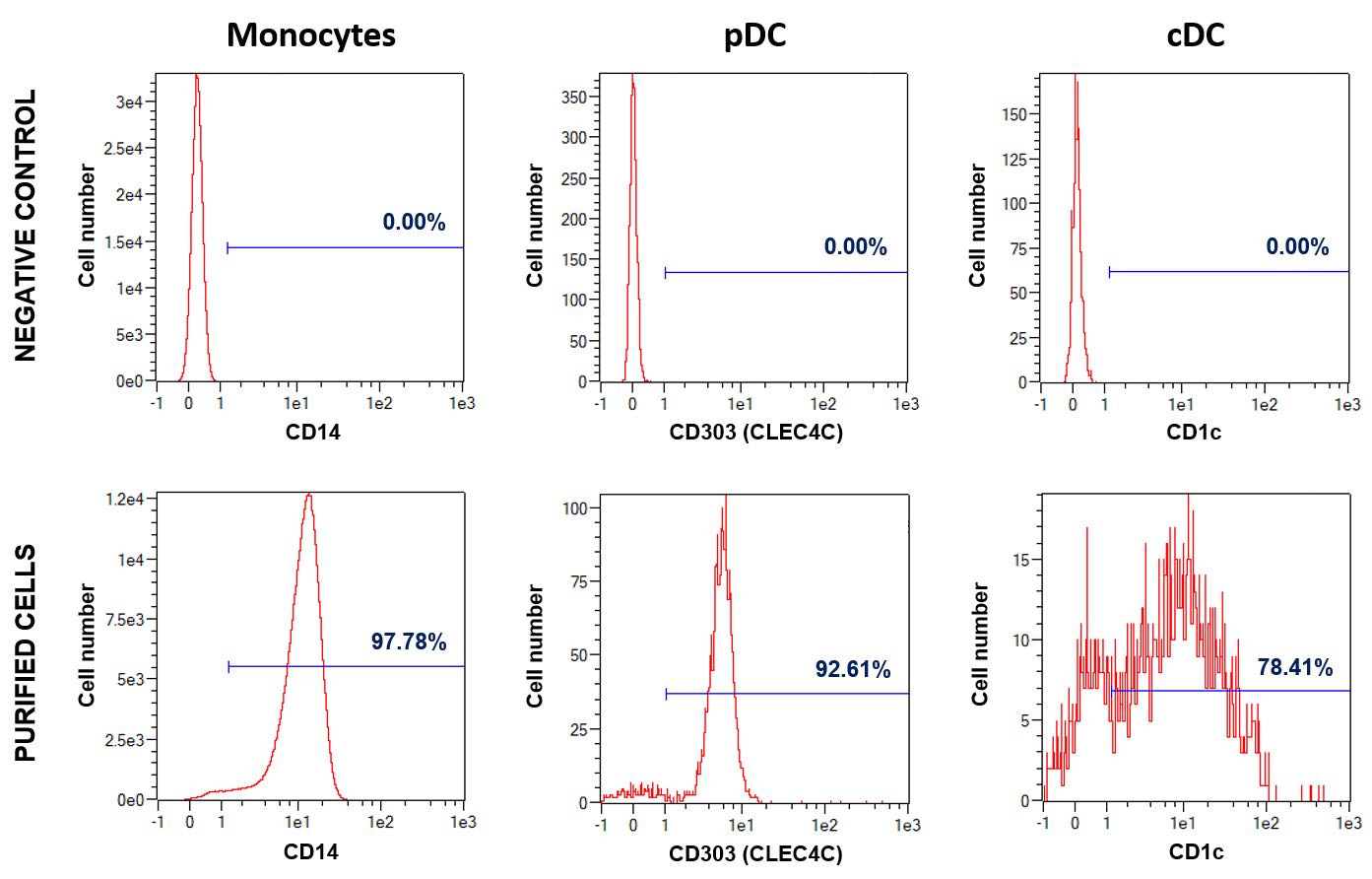


**Supplementary Figure S1.** Representative flow cytometry histograms with percentage of positive cells obtained from purified (bottom row) CD14^+^ monocytes, plasmacytoid dendritic cells (pDC), and conventional dendritic cells (cDC). Cells were stained with specific surface markers using CD14, CD303 (CLEC4C) and CD1c antibodies correspondingly, or negative control (top row). Negative control (upper row) antibodies used, were isotype control IgG1-PE antibody for CD14 and isotype control human IgG1-AP for CD1c and CD303.


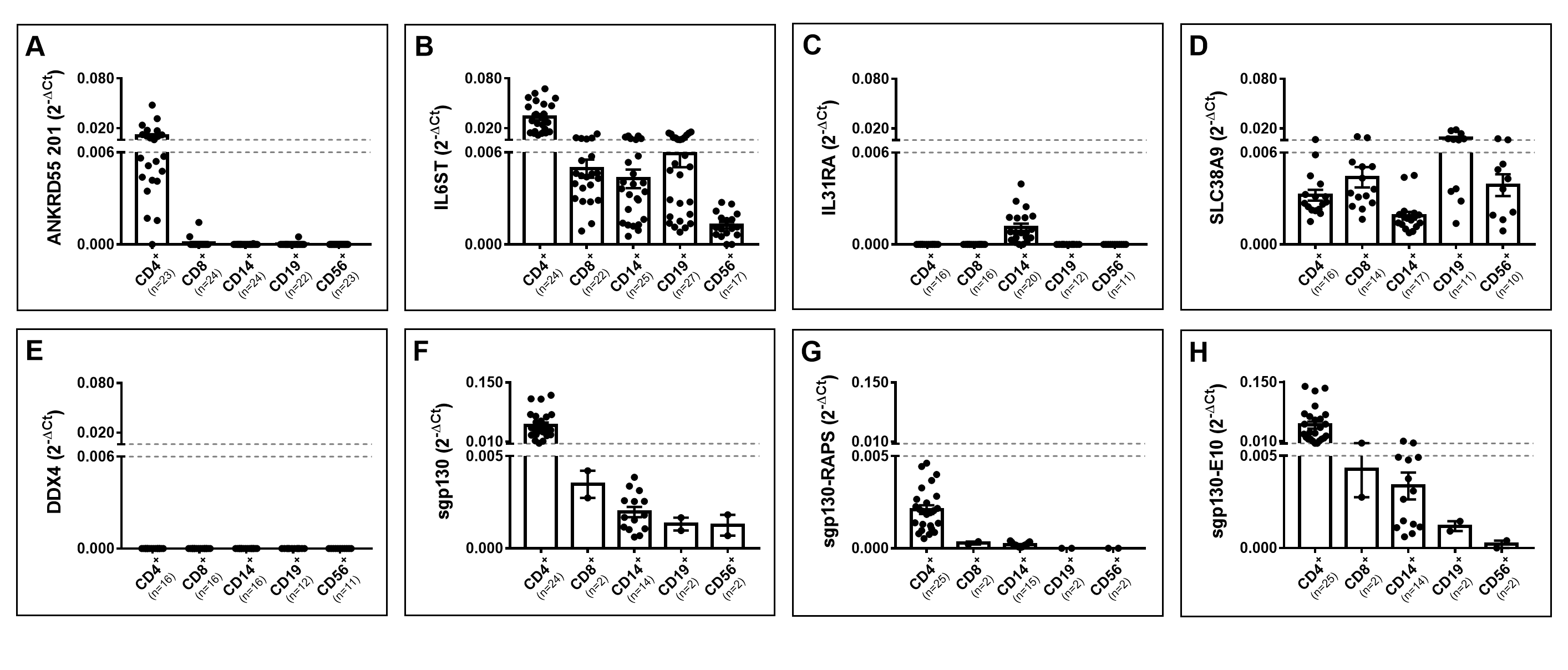


**Supplementary Figure S2.** Gene expression by qPCR of **(A)** *ANKRD55* main isoform 201, **(B)** *IL6ST*, **(C)** *IL31RA*, **(D)** *SLC38A9*, **(E)** *DDX4*, **(F)** sgp130, **(G)** sgp130-RAPS, **(H)** sgp130-E1 in CD4^+^, CD8^+^, CD14^+^, CD19^+^, and CD56^+^ subpopulations of PBMCs from healthy controls. **(A-H)** Data are mean ± SEM; numbers of different healthy controls used in each condition are indicated.

**
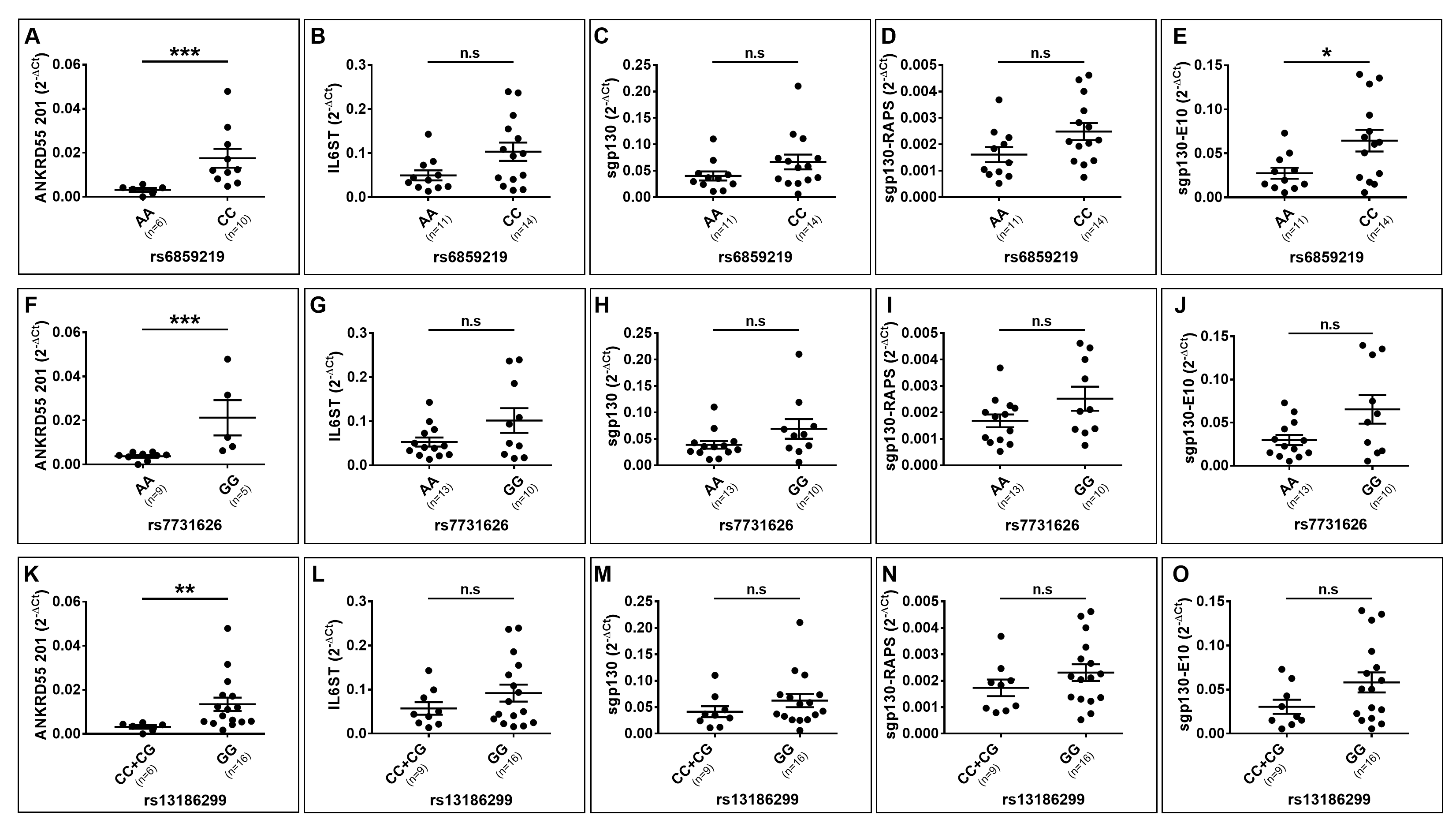
**

**Supplementary Figure S3.** Effect of homozygosity for protective or risk alleles of the MS risk SNPs **(A-E)** rs6859219, **(F-J)** rs7731626 and **(K-O)** of the correlated SNP rs13186299 on expression of *ANKRD55* isoform 201, *IL6ST*, sgp130, sgp120-RAPS and sgp130-E10 as measured by qPCR in CD4^+^ T cells from healthy controls. The risk genotypes are CC (rs6859219), GG (rs7731626) and GG (rs13186299). Data are mean ± SEM; the numbers of individual subjects analyzed per genotype are indicated between brackets; Mann-Whitney test.

**
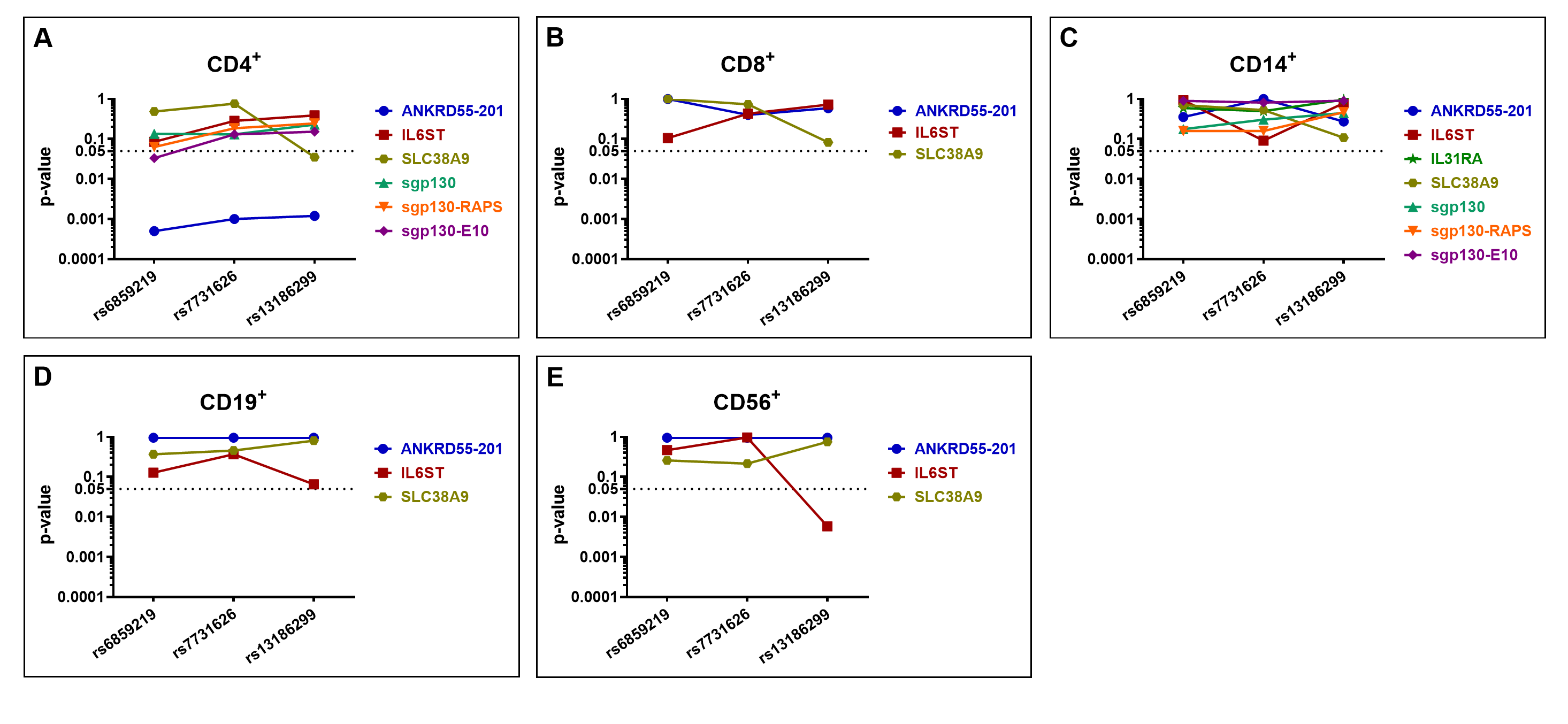
**

**Supplementary Figure S4.** Plot of the *p* values for association of MS risk SNP genotype with expression levels of *ANKRD55*, *IL6ST* and *SLC38A9* in CD4^+^, CD8^+^, CD14^+^, CD19^+^ and CD56^+^ cells from healthy controls. *IL6ST* isoforms sgp130, sgp130-RAPS and sgp130-E10 were only analyzed in CD4^+^ and CD14^+^ cells, and *IL31RA* in CD14*^+^* cells. Mann-Whitney test.

**
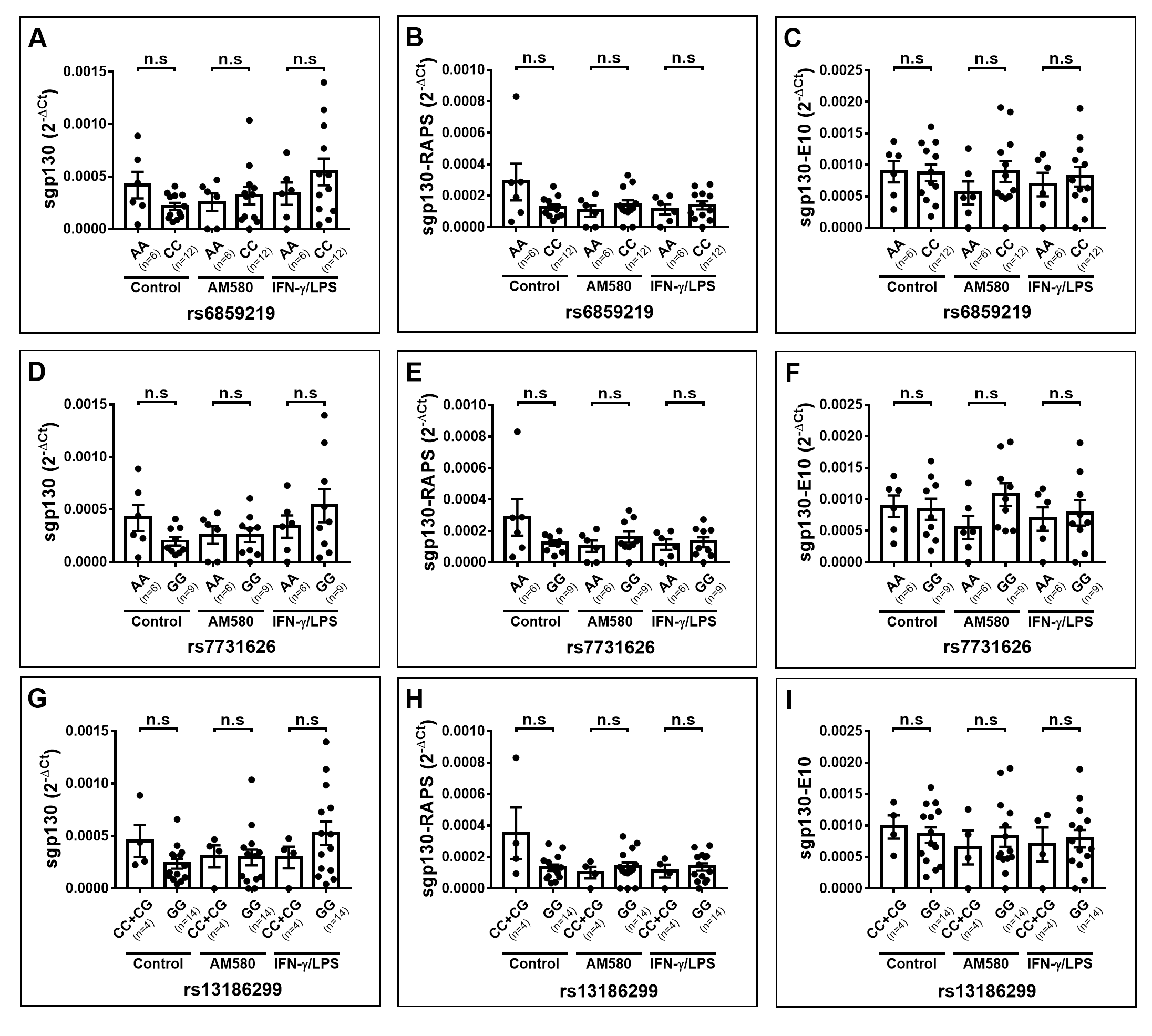
**

**Supplementary Figure S5.** Effect of homozygosity for protective or risk alleles of the MS risk SNPs **(A-C)** rs6859219, **(D-F)** rs7731626 and **(G-I)** correlated SNP rs13186299 on expression of sgp130, sgp130-RAPS and sgp130-E10 in immature, AM580-treated or IFN-γ/LPS-matured moDC of healthy donors measured by qPCR. Monocytes were co-cultured in MoDC medium for 6 days in the presence or absence (Control) of 100 nM AM580 from day 0 and new AM580 was added on day 3 of the moDC differentiation period. Alternatively, moDC were matured with a combination of 0.5 pg/ml IFN-γ for 12 hours and 200 ng/ml LPS for 6 hours. The risk genotypes are CC (rs6859219), GG (rs7731626) and GG (rs13186299). Data are mean ± SEM; the numbers of individual subjects analyzed per condition are indicated between brackets; Mann-Whitney test or unpaired *t*-test.

**Supplementary Table S1: Clinical data of MS patients**

|  |  | N | %women/%men | Age (years)^a^ | Duration of  disease (years)^a^ | EDSS^b^ |
| --- | --- | --- | --- | --- | --- | --- |
|  | PP | 16 | 56.25/43.75 | 54.75 (±10.98) | 12.49 (±6.80) | 5.25 (4.00-6.50) |
| **Cohort 1^c^** | RR | 19 | 63.16/36.84 | 35.63 (±8.85) | 3.21 (±4.62) | 0.00 (0.00-2.00) |
|  | SP | - | - | - | - | - |
|  | PP | 28 | 39.29/60.71 | 51.50 (±9.10) | 12.59 (±10.32) | 5.50 (3.50-6.00) |
| **Cohort 2^d^** | RR | 11 | 81.82/18.18 | 39.00 (±8.66) | 11.18 (±9.86) | 2.50 (1.50-4.00) |
|  | SP | 4 | 75.00/25.00 | 44.50 (±4.20) | 10.67 (±10.18) | 3.50 (3.00-4.38) |
|  | PP | 10 | 50.00/50.00 | 56.70 (±9.55) | 15.38 (±12.58) | 6.00 (2.38-6.63) |
| **Cohort 3^e^** | RR | 40 | 70.00/30.00 | 38.08 (±11.48) | 4.46 (±5.67) | 2.00 (1.50-2.00 |
|  | SP | - | - | - | - | - |

^a^Data are expressed as mean (±standard deviation); ^b^Data are expressed as median (Quartile1-Quartile3). ^c^Cohort 1 = IIS Biocruces Bizkaia MS Cohort, Barakaldo; ^d^Cohort 2 = Hospital Universitari Vall d’Hebron MS Cohort, Barcelona, ^e^Cohort 3 = Hospital Universitario Ramón y Cajal MS Cohort, Madrid.

**Supplementary Table S2: Primers used in the study**

| **Application** | **Gene** | **Forward primer**  **(5’-3’)** | **Reverse primer**  **(5’-3’)** | **Reference** |
| --- | --- | --- | --- | --- |
| qPCR | *ACTB_1* | - | - | Predesigned  (QIAGEN, Ref. Hs_ACTB_1_SG) |
|  | *ACTB_2* | - | - | Predesigned  (QIAGEN, Ref. Hs_ACTB_2_SG) |
|  | *GAPDH* | GCAACAATATCCACTTTACCAGAG | CACATCGCTCAGACACCAT | Own design |
|  | *ANKRD55*  *(best coverage)* | - | - | Predesigned  (IDT, Ref. Hs.PT.58.27501603) |
|  | *ANKRD55_201* | CAGCCTCAACACACACAAATGC | TAGTTGATTATGGACGGCCCCTG | Ref. 1 |
|  | *ANKRD55_202* | CGGGCTCATTTAACACTTACTATTTC | CTTAGCCAGCAACAGCTCCTG | Ref. 1 |
|  | *ANKRD55_204* | TCCACTATGCTCGGCTGC | CTCGCTGATGTTCGACTGTTG | Ref. 1 |
|  | *IL6ST* | CCGCCACATAATTTATCAGT | AAGGTCTTGGACAGTGAATG | Ref. 2 |
|  | *IL31RA* | CTCTGCGATGTGCGGTCAA | GCCAAGTGTTTTCTCTAGGACTG | Own design |
|  | *SLC38A9* | TCCAGCTCCAGAAGAGTGCT | CTGTTTTATGCCCCAAGGAA | Own design |
|  | *DDX4* | TCATACTTGCAGGACGAGATTTG | AACGACTGGCAGTTATTCCATC | Own design |
|  | sgp130 | GCAGCATACACAGATGAAGGTG | TAAGCTGTAAGGTCCTCGTTGG | Ref. 3 |
|  | sgp130-RAPS | TCCACCCGATCTTCATTCACTG | AGGAGGCAATGTTATCTTCATAGG | Ref. 3* |
|  | sgp130-E10 | TCCATCCCATACTCAAGGCTAC | TCACAGATACAAACCTTGAAAGTCAC | Ref. 3 |
|  | *ALDH1A2* | GAGGAGTTTGTGAGAAGAAGCGT | CTGTGGGCTCAATGAAAAACC | Ref. 4 |
|  | *RARA* | AGATTACTGACCTGCGAAGC | CTCTGAGTTCTCCAACATTTCCT | Predesigned  (IDT, Ref. Hs.PT.58.442330) |
|  | *RARG* | AACAAGGTGACCAGGAATCG | CTGTCAGGTGACCCTTCTTC | Predesigned  (IDT, Ref. Hs.PT.58.38473235) |
| ddPCR | *ANKRD55_201* | - | - | Predesigned  (Bio-Rad, Ref. dCNS104464519) |
|  | *IL6ST* | - | - | Predesigned  (Bio-Rad, Ref. dHsaCPE5047372) |
|  | *IL31RA* | - | - | Predesigned  (Bio-Rad, Ref. dHsaCPE5052967) |
|  | *SLC38A9* | - | - | Predesigned  (Bio-Rad, Ref. dHsaCPE5057630) |
|  | *IL3RA* | - | - | Predesigned  (Bio-Rad, Ref. dHsaCPE5058092) |
|  | *CD1C* | - | - | Predesigned  (Bio-Rad, Ref. dHsaCPE5038928) |
|  | *CLEC4C* | - | - | Predesigned  (Bio-Rad, Ref. dHsaCPE5048022) |

Ref. 1: “Novel insights into the multiple sclerosis risk gene ANKRD55”. De Lapuente AL, *J Immunol* (2016) 196(11):4553–65. doi: 10.4049/jimmunol.1501205.

Ref. 2: “Oncostatin M Secreted by Skin Infiltrating T Lymphocytes Is a Potent Keratinocyte Activator Involved in Skin Inflammation”. Boniface K*, J. Immunol* (2007) 178(7):4615-4622. doi: 10.4049/jimmunol.178.7.4615.

Ref. 3: “Different Soluble Forms of the Interleukin-6 Family Signal Transducer gp130 Fine-Tune the Blockade of Interleukin-6 Trans-Signaling”. Wolf J, *J Biol Chem* (2016) 291(13):16186-16196. doi: 10.1074/jbc.M116.718551.

(*) Reverse primer: first and last base from original sequence were deleted to improve primer characteristics.

Ref. 4: “Interleukin-22 binding protein (IL-22BP) is constitutively expressed by a subset of conventional dendritic cells and is strongly induced by retinoic acid”. Martin JCJ., *Mucosal Immunol* (2014) 7(1).101-113. doi: 10.1038/mi.2013.28.

**Supplementary Table S3. Effect of risk SNP homozygosity on AM580-induced expression of *ANKRD55* isoforms by qPCR**

| **Isoform** | **SNP** | **Genotype** | **Average fold change (AM580/Control)** | ***p*-value** | **Number of**  **paired samples** |
| --- | --- | --- | --- | --- | --- |
| *ANKRD55-201* | rs6859219 | AA | 1,55 | 0,1875 | 5 |
|  |  | CC | 3,32 | 0,0020 (**) | 11 |
|  | rs7731626 | AA | 1,55 | 0,1875 | 5 |
|  |  | GG | 3,12 | 0,0156 (*) | 7 |
|  | rs13186299 | CG | 1,19 | 0,7500 | 3 |
|  |  | GG | 3,21 | 0,0010 (***) | 12 |
| *ANKRD55-202* | rs6859219 | AA | 1,38 | 0,6250 | 5 |
|  |  | CC | 2,56 | 0,0049 (**) | 11 |
|  | rs7731626 | AA | 1,38 | 0,6250 | 5 |
|  |  | GG | 2,64 | 0,0156 (*) | 7 |
|  | rs13186299 | CG | 1,26 | >0,9999 | 3 |
|  |  | GG | 2,51 | 0,0024 (**) | 12 |
| *ANKRD55-204* | rs6859219 | AA | 2,31 | 0,0625 | 5 |
|  |  | CC | 2,89 | 0,0010 (***) | 11 |
|  | rs7731626 | AA | 2,31 | 0,0625 | 5 |
|  |  | GG | 2,75 | 0,0156 (*) | 7 |
|  | rs13186299 | CG | 2,38 | 0,2500 | 3 |
|  |  | GG | 2,79 | 0,0005 (***) | 12 |
